# The neural system of metacognition accompanying decision-making in the prefrontal cortex

**DOI:** 10.1371/journal.pbio.2004037

**Published:** 2018-04-23

**Authors:** Lirong Qiu, Jie Su, Yinmei Ni, Yang Bai, Xuesong Zhang, Xiaoli Li, Xiaohong Wan

**Affiliations:** State Key Laboratory of Cognitive Neuroscience and Learning and IDG/McGovern Institute for Brain Research, Beijing Normal University, Beijing, China; University of Oxford, United Kingdom of Great Britain and Northern Ireland

## Abstract

Decision-making is usually accompanied by metacognition, through which a decision maker monitors uncertainty regarding a decision and may then consequently revise the decision. These metacognitive processes can occur prior to or in the absence of feedback. However, the neural mechanisms of metacognition remain controversial. One theory proposes an independent neural system for metacognition in the prefrontal cortex (PFC); the other, that metacognitive processes coincide and overlap with the systems used for the decision-making process per se. In this study, we devised a novel “decision–redecision” paradigm to investigate the neural metacognitive processes involved in redecision as compared to the initial decision-making process. The participants underwent a perceptual decision-making task and a rule-based decision-making task during functional magnetic resonance imaging (fMRI). We found that the anterior PFC, including the dorsal anterior cingulate cortex (dACC) and lateral frontopolar cortex (lFPC), were more extensively activated after the initial decision. The dACC activity in redecision positively scaled with decision uncertainty and correlated with individual metacognitive uncertainty monitoring abilities—commonly occurring in both tasks—indicating that the dACC was specifically involved in decision uncertainty monitoring. In contrast, the lFPC activity seen in redecision processing was scaled with decision uncertainty reduction and correlated with individual accuracy changes—positively in the rule-based decision-making task and negatively in the perceptual decision-making task. Our results show that the lFPC was specifically involved in metacognitive control of decision adjustment and was subject to different control demands of the tasks. Therefore, our findings support that a separate neural system in the PFC is essentially involved in metacognition and further, that functions of the PFC in metacognition are dissociable.

## Author summary

Decision-making is often accompanied by a sense of uncertainty regarding the outcome. In many situations, there is no explicit feedback or cue to indicate whether the decision is correct or not. Fortunately, our brain can evaluate decision uncertainty using the internal signals and subsequently make appropriate adjustments to initial decisions. The process of considering the outcome of a decision and whether a decision should be adjusted is called metacognition, and it tends to be automatically induced. Thus, decision-making is usually accompanied by metacognition, and the two processes are inevitably coupled. However, the neural systems supporting metacognitive processing remain unclear and have often been misattributed to the neural system of the decision-making process per se. Here, we have analyzed this process in several volunteers by imaging the brain activity in specific regions while they performed Sudoku and random-dot motion (RDM) tasks. Our results suggest the existence of a neural system located in the prefrontal cortex (PFC) mainly involved in metacognition and independent from the neural system of decision-making.

## Introduction

Decision-making is a process of evidence accumulation. That evidence may come from sensory signals of external stimuli or from mental representations of internal cognitive operations. Variations in evidence can create uncertainty in the person rendering a decision. The decision maker is normally explicitly or implicitly aware of uncertainties about a decision and consequently confirms or revises a decision even prior to, or in the absence of, external feedback. In the framework of cognitive control, the processes of decision uncertainty monitoring—and consequent decision adjustments—are termed metacognition, that is, ‘cognition about cognition’ [1–4]. Although metacognition generally accompanies decision-making with uncertainty, the underlying neural system of the metacognitive processes in decision uncertainty monitoring and consequent decision adjustments remains less clear than that of the decision-making process per se [5, 6].

Much of the work on the neural bases of metacognition in humans has focused on metacognitive monitoring of internal states (i.e., confidence or uncertainty) with regard to the cognitive processes such as episodic memory [7, 8] and sensory perception [9, 10]. Behaviorally, confidence ratings, which reflect subjective accuracy beliefs regarding decisions, have often been found to deviate from the accuracy of an actual decision [11–13]. These observations have suggested the existence of a separate neural processing system (meta-level) in the generation of decision confidence or uncertainty, independent of the decision-making process per se (object-level). We hereafter refer to this description of metacognition as separable from decision-making as “Theory 1” [11–18]. The prefrontal cortex (PFC) has been proposed to play a critical role in metacognition [14], and it has been demonstrated that interference with or lesions in PFC regions may impair metacognitive monitoring of perceptual decisions, but not decisions per se [15–18, but see also 19].

A contrary theory, which we will refer to as “Theory 2,” suggests that metacognition may be merely dependent on the decision-making process and therefore exclusively reliant on accumulated evidence [20–24]. Specifically, this theory, based on bounded accumulation models, has interpreted divergence between decision accuracy and confidence reports as being caused by the accumulation of postdecisional evidence during the interval between decision-making and confidence reporting [20–24]. Furthermore, it implies that decision adjustment naturally occurs as a part of this continuous postdecisional evidence accumulation and therefore is an integrated part of the initial decision-making process [21, 24]. Some proponents of this theory have argued that a separate neural system for metacognition to monitor and control decision-making should not be necessary because the processes are interdependent [24]; however, not all work supporting this theory insists on this notion [20].

**Fig 1:**
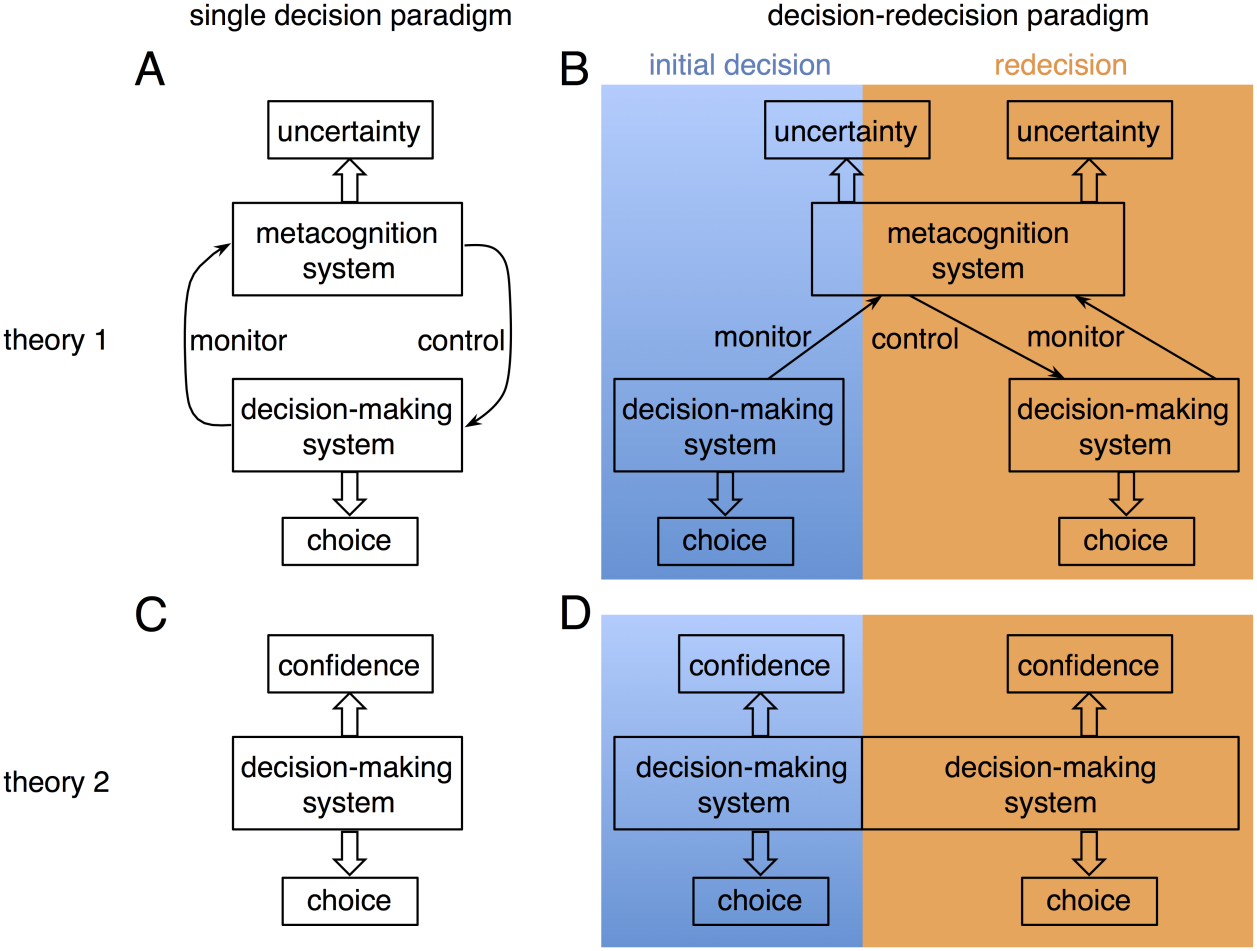
Scheme of decisional processing in single-decision and decision–redecision task paradigms. (Panel A and B—Theory 1). (A) Although the decision-making system and the metacognition system are separate, the metacognition system monitors and controls ongoing decisional processes in a single-decision paradigm. The choice is determined by the decision-making system, and the decision uncertainty is encoded in the metacognition system. (B) In the decision–redecision paradigms, the metacognition system monitors the initial decisional process and continuously both monitors and controls proceeding decisional processing throughout consecutive decision-making processes involved in redecision. (Panel C and D—Theory 2). Theory 2 proposes that decision-making processes and metacognitive processes share a single underlying neural system. (C) The choice and the decision confidence are both outcomes of the same underlying neural system in the single-decision task paradigm. (D) In the decision–redecision paradigm, the same underlying system also supports decision-making and metacognition both for the initial decision as well as in redecision.

Thus, one of the crucial issues in the debate between the two theories is whether a separate neural system for metacognition exists. Single-decision paradigms (depicted in Fig 1A and 1C) are not sufficient to determine the existence or nonexistence of separable systems because the decision-making process and the metacognitive process are inevitably coupled in such tasks. The purpose of retrospective metacognition is to confirm or revise foregone decisions. Given an opportunity to make a decision on the same situation again (i.e., make a “redecision”) (as depicted in tasks shown in Fig 1B and 1D), a decision maker may revise an initial decision as well as confidence in the decision once s/he detects uncertainty regarding the initial decision [25]. Thus, if a separate neural system for metacognition exists as proposed by Theory 1, the metacognitive processes—in particular metacognitive control—should be more extensively involved in redecision, especially if the initial decision must be made quickly. On the contrary, the neural system involved in redecision should be the same as those involved in an initial decision if there is not a separate neural system for metacognition (as proposed by Theory 2). If a separate neural system for metacognition exists, the activity of this system should be manifest after an initial decision is reached, whereas Theory 2 suggests that they share the same underlying neural systems and that neural activity following either a single decision or a redecision should be the same.

Therefore, comparing behavioral and neural differences between the two phases of initial decision and redecision may allow us to test which theory better accounts for the neural processing of metacognition. A specific perspective of metacognition derived from Theory 1 implies that decision uncertainty, rather than decision confidence, should be the key signal for metacognition. If there is no uncertainty regarding a decision, it should not evoke the processes of metacognitive monitoring and control. Therefore, the critical aim of this study was to elicit and analyze neural activity positively correlated with decision uncertainty, rather than that positively correlated with decision confidence.

**Fig 2:**
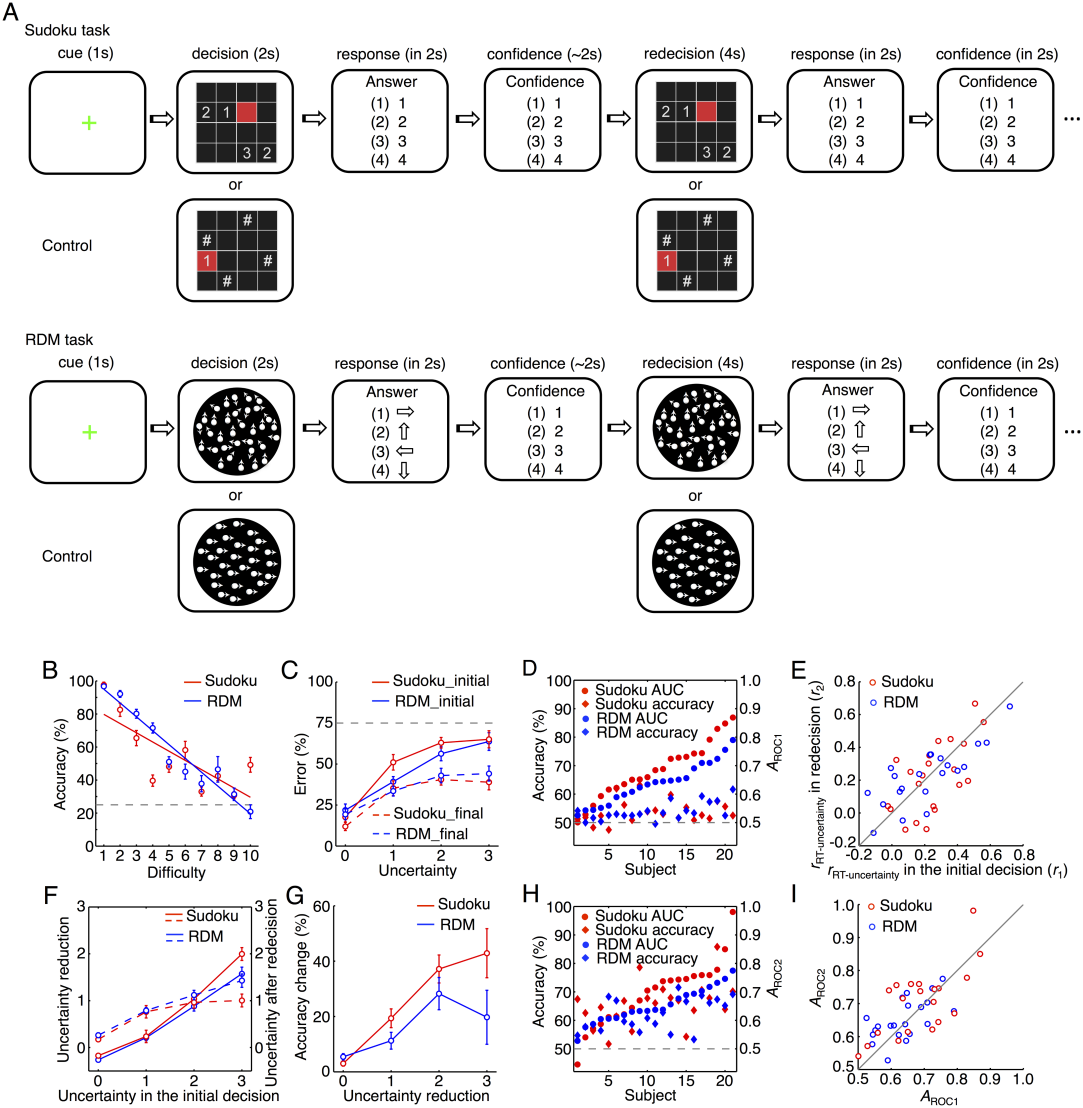
Experimental paradigms and behavioral performance in the two tasks. (A) The Sudoku and RDM task sequences. (B) The relationship between task difficulty and the mean accuracy in the initial decision (2-s task immediately after training). (C) The relationship between the decision uncertainty level of the initial decision (1–4 confidence ratings) and the incorrect percentage in the initial and final decisions. (D) The individual uncertainty sensitivity (*A*_ROC_, circles) and decision accuracy (diamonds) in the initial decision. (E) The individual *r*_RT-uncertainty_ (correlation coefficient between RT and decision uncertainty) in the initial and final decisions. (F) The relationship between the decision uncertainty level of the initial decision and the extent of uncertainty reduction by redecision (solid lines) and the decision uncertainty level after redecision (broken lines). (G) The relationship between the extent of decision uncertainty reduction and the accuracy change by redecision. (H) The individual uncertainty sensitivity (*A*_ROC_, circles) and decision accuracy (diamonds) in the final decision. (I) The individual uncertainty sensitivity (*A*_ROC_) in the initial and final decisions. The data illustrated from C–I were from the main fMRI experiment (fMRI1). Red, the Sudoku task; blue, the RDM task. Error bars indicate SEM across the participants. The data can be found in S1 Data. AUC, area under curve; fMRI, functional magnetic resonance imaging; RDM, random-dot motion; RT, response time.

In the present study, we employed a novel “decision–redecision” experimental paradigm to investigate neural activity associated with metacognition. The participants were asked to make two consecutive decisions on the same situation using a perceptual decision-making task and a rule-based decision-making task (Fig 2A). We combined this novel paradigm with the functional magnetic resonance imaging (fMRI) technique to formally test the two theories and systematically investigate the underlying neural substrates of metacognitive processes accompanying decision-making. Based on our previous study [25], we expected that the frontoparietal control network would be associated with metacognitive processing. In the current study, we focused on specific functions of the regions in the network believed to be involved in metacognition. We found that dorsal anterior cingulate cortex (dACC) activity significantly correlated with metacognitive monitoring of decision uncertainty and that lateral frontopolar cortex (lFPC) activation correlated instead with metacognitive control. These findings provide evidence for distinct neural processes involved in metacognition and decision-making.

## Results

### Task paradigm

We developed a novel decision–redecision paradigm for this study (Fig 2A). The participants made an initial decision (decision phase), immediately followed by another decision on the same situation (redecision phase). This allowed the participant the opportunity to revise the initial decision and update their confidence rating, even without feedback. The internal states of uncertainty regarding initial and final decisions were separately evaluated by confidence ratings. Confidence was rated on a scale of 1 to 4, immediately following the corresponding decisions. Decision uncertainty was then the inverse of the confidence rating (i.e., a confidence rating of 4 corresponded to an uncertainty rating of 1). Critically, our task differed from previous paradigms used to analyze ‘change of mind’ [21, 24]. Previous task paradigms were only able to analyze the small portion of trials in which a participant happened to change their mind, while our paradigm allowed analysis of each trial.

We used two different types of decision-making tasks in the present study: one was a rule-based decision-making task (Sudoku), the other a perceptual decision-making task (random-dot motion [RDM]), which has commonly been used to investigate the neural process of decision-making [5] and more recently, metacognition [21, 22, 24, 26]. The decisions in the Sudoku task rely on internal informational operations, but decisions in the RDM task should be more dependent on accumulation of external information. It is possible to continue accumulating evidence from external stimuli that may affect decision-making in the RDM task, but that is less likely in the Sudoku task because it is rule based. For this reason, the two tasks should result in differential processing in metacognitive control to adjust initial decisions. The sequences of both tasks were identical (Fig 2A, illustrated for the main fMRI experiment [fMRI1]). After a Sudoku problem or RDM stimulus was presented for 2 s, the participant made a choice from 4 possible solutions within 2 s and then reported their confidence rating on that decision within 2 s. A critical feature of our paradigm was that the same Sudoku problem or the same RDM stimulus was immediately repeated for 4 s, and the participant again made a choice within 2 s and again reported their confidence rating within 2 s. To better distinguish the metacognitive process from the decision-making process, we intentionally set a short initial decision phase (2 s), to minimize metacognition during the initial decision-making phase, but set a longer duration in the redecision phase (4 s) to allow enough time for metacognitive processing in redecision. There was no explicit feedback or cue to indicate whether the decision was correct after either the initial decision or the redecision. For both tasks, the task difficulty of each trial (Fig 2B) was adaptively adjusted by a staircase procedure [9, 27] so that the average accuracy for the initial decision was converged to approximately 50% (chance level was 25%). For the control condition, the participant was shown a digital number in the target grid in the Sudoku task, and for the RDM task, s/he was shown an RDM stimulus with 100% coherence. For the former, the participant only needed to press the button matching the number, and for the latter, the participant indicated the unambiguous RDM direction. Prior to the experimental testing, the participant was trained to attain high-level proficiency in Sudoku problem-solving.

The current study was composed of 4 fMRI experiments:

- fMRI1 was the main experiment to illustrate the neural system of metacognition accompanying decision-making.
- fMRI2 was designed to test whether the neural system of metacognition would also be activated during the initial decision-making when a new Sudoku problem or a new RDM stimulus was presented in the redecision phase.
- fMRI3 was designed to test whether redecision activated the neural system of metacognition more strongly than the condition with no redecision because redecision should involve additional neural processing for metacognitive control.
- fMRI4 was designed to confirm that the engagement of metacognition was independent of the duration of redecision (from 4 s in fMRI1 to 2 s in fMRI4 in the RDM task).

### Behavioral results

Twenty-one participants took part in fMRI1 (see Materials and methods). In both the Sudoku and RDM tasks, decision uncertainty levels were largely consistent with the percentage of incorrect initial decisions (Fig 2C; Pearson’s *r* = 0.76 ± 0.12 [mean ± SD], one-tailed *t* test, *t*_21_ = 7.3, *P* = 1.7 × 10^−7^ in the Sudoku task; *r* = 0.71 ± 0.14, *t*_21_ = 6.8, *P* = 5.0 × 10^−7^ in the RDM task). To examine the trial-by-trial consistency between objective erroneous decisions and subjective decision uncertainty levels in individual participants, a nonparametric approach was employed to construct the receiver operating characteristic (ROC) curve by using the decision uncertainty levels as thresholds to characterize the likelihood of erroneous decisions. The area under curve (*A*_ROC_) was then calculated to represent the individual uncertainty sensitivity, indicating how sensitive the participant was to the decision uncertainty [9]. As observed in the previous studies, the uncertainty sensitivity of individual participants markedly deviated from decision accuracy in both tasks, which were controlled around 50% (Fig 2D). The response times (RTs) of option choices in the initial decision were positively correlated with the decision uncertainty levels (Fig 2E; *t*_21_ = 6.9, *P* = 4.0 × 10^−7^ in the Sudoku task; *t*_21_ = 4.3, *P* = 1.6 × 10^−4^ in the RDM task). The correlation coefficient between RT of option choices and the decision uncertainty level (*r*_RT-uncertainty_) in the initial decision was highly correlated with the uncertainty sensitivity (*A*_ROC1_) across the participants (Pearson’s *r* = 0.61, *z* test, *z* = 3.4, *P* = 4.0 × 10^−4^ in the Sudoku task; *r* = 0.48, *z* = 2.4, *P* = 0.0085 in the RDM task). Thus, the RT–uncertainty correlation also reflected individual uncertainty sensitivity.

The level of decision uncertainty was reduced by redecision. The extent of decision uncertainty reduction via redecision was highly correlated with the decision uncertainty level in the initial decision phase (Fig 2F; Goodman and Kruskal’s *γ* = 0.82 ± 0.11, *t*_21_ = 8.8, *P* = 2.1 × 10^−8^ in the Sudoku task; *γ* = 0.78 ± 0.14, *t*_21_ = 7.7, *P* = 8.2 × 10^−8^ in the RDM task). Accordingly, accuracy also improved along with uncertainty reduction (Fig 2G; Pearson’s *r* = 0.54 ± 0.13, *t*_21_ = 4.2, *P* = 2.3 × 10^−4^ in the Sudoku task; *r* = 0.39 ± 0.14, *t*_21_ = 2.8, *P* = 5.6 × 10^−3^ in the RDM task). One could suspect that the improvement of uncertainty reduction and the change in accuracy in the redecision phase were caused by regression towards mean in the two separate decisions: higher uncertainty at the first measurement by chance would increase improvement at the second measurement. However, the decision accuracy and decision uncertainty levels for the final decision-making phase remained significantly differential (Pearson’s *r* = 0.35 ± 0.15, *t*_21_ = 2.1, *P* = 0.032 in the Sudoku task; *r* = 0.36 ± 0.14, *t*_21_ = 2.6, *P* = 8.9 × 10^−3^ in the RDM task in Fig 2C; Pearson’s *r* = 0.32 ± 0.14, *t*_21_ = 2.0, *P* = 0.042 in the Sudoku task; *r* = 0.32 ± 0.15, *t*_21_ = 2.2, *P* = 0.028 in the RDM task in Fig 2G), indicating that the participants’ performance in redecision reflected metacognitive processing ability rather than chance. Despite the fact that both decision accuracy and decision uncertainty levels were improved in the redecision phase, the divergence between uncertainty sensitivity and decision accuracy remained significant (Fig 2H). Indeed, neither the individual uncertainty sensitivities nor those of individual differences were altered by redecision (Fig 2I; *t*_21_ = 0.82, *P* = 0.21 in the Sudoku task; *t*_21_ = 1.0, *P* = 0.15 in the RDM task). Similarly, neither the individual RT–uncertainty correlation coefficients nor those of individual differences were altered by redecision (Fig 2E; *t*_21_ = −0.77, *P* = 0.22 in the Sudoku task; *t*_21_ = 0.35, *P* = 0.36 in the RDM task). These results show that individual uncertainty sensitivity was stable, was intrinsic to individual metacognitive ability, and was independent of the accumulated evidence and the type of decision-making required.

### Neural correlates of metacognitive monitoring and control in redecision processing

**Fig 3:**
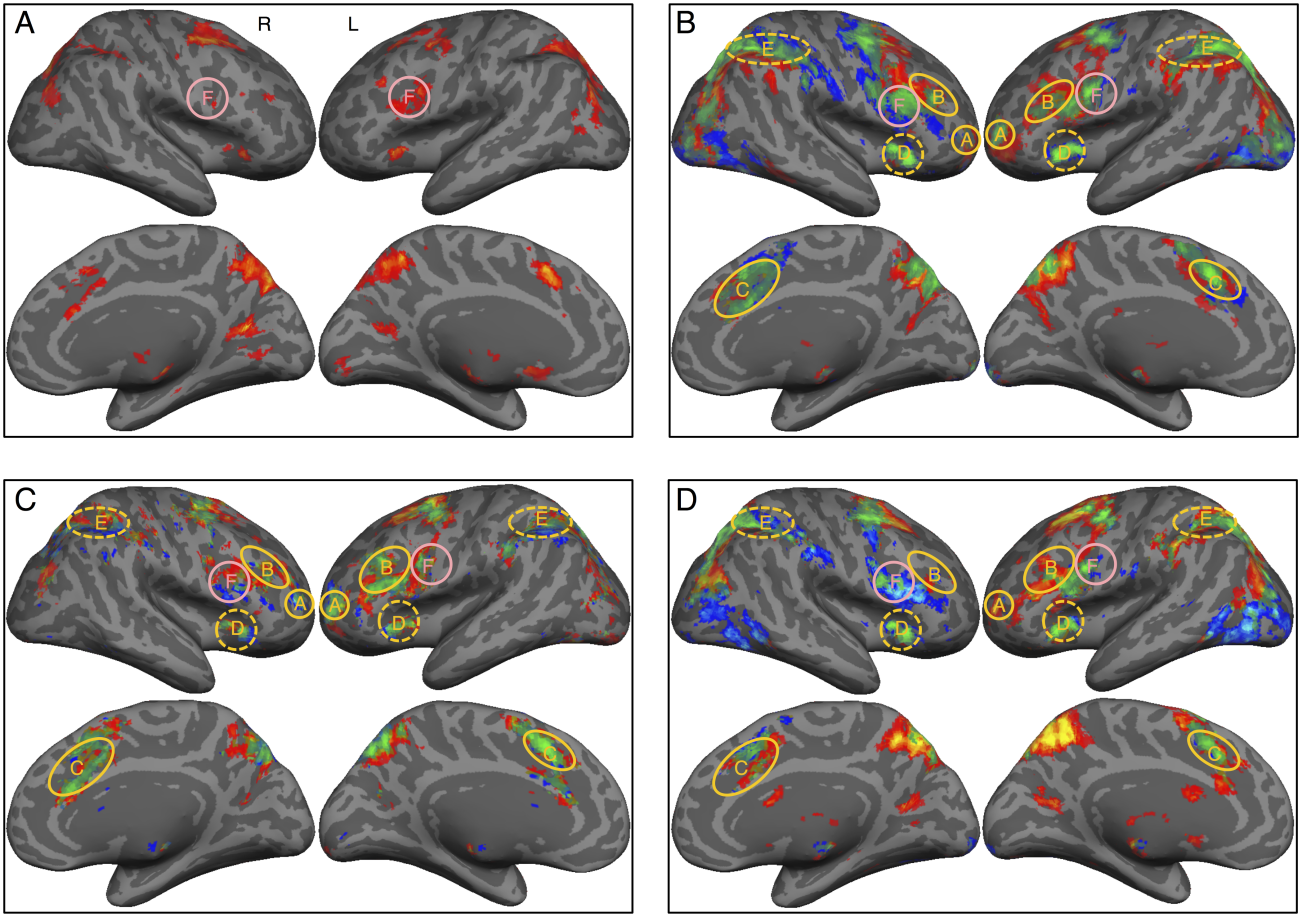
The neural network involved in metacognitive monitoring and control. (A) Activation in the task trials compared with the control trials during the initial decision phase (significant only in Sudoku task). (B) Activation in the task trials compared with the control trials during the redecision phase. (C) Activation in task trials during the redecision phase regressed with decision uncertainty levels. (D) Activation in task trials with redecision required the ‘redecision’ condition, compared with the “non-redecision” condition in fMRI3. Red/yellow patches indicate activations in the Sudoku task, blue/light-blue patches indicate activations in the RDM task; *z* = 3.1, *P* < 0.05, FDR-corrected. Green/light-green patches indicate conjunction activations across the two tasks; *z* = 3.1, *P* < 0.001, cluster-size corrected. “A” denotes the lFPC; “B” denotes the mDLPFC; “C” denotes the dACC; “D” denotes the AIC; “E” denotes the aIPL; and “F” denotes the IFJ. AIC, anterior insular cortex; aIPL, anterior inferior parietal lobule; dACC, dorsal anterior cingulate cortex; FDR, false discovery rate; fMRI, functional magnetic resonance; IFJ, inferior frontal junction; lFPC, lateral frontopolar cortex; mDLPFC, middle dorsolateral prefrontal cortex; RDM, random-dot motion.

Commonly across both tasks, brain activation during the initial decision phase was mainly restricted to brain areas posterior to the PFC, in particular the posterior portion of the PFC, the inferior frontal junction (IFJ) (S1A Fig; Fig 3A). In the redecision phase, a frontoparietal control network—consisting of the lFPC, dACC, anterior insular cortex (AIC), middle dorsolateral PFC (mDLPFC), and anterior inferior parietal lobule (aIPL)—was more extensively recruited (Fig 3B; S1B and S2 Figs; S1 Table). In contrast, the lFPC and mDLPFC regions of the frontoparietal control network were not activated when a new Sudoku problem or a new RDM stimulus was presented for the first time during the redecision phase, preceded by the control stimuli in the initial phase (fMRI2, *n* = 17; S1C and S3 Figs), while the dACC activity during the same phase became much weaker, and its response onset was much delayed from the onset of the stimulus presentation (delay offset >3 s; S3 Fig). Thus, the frontoparietal control network, in particular the regions of the lFPC, mDLPFC, and dACC in the anterior FPC, were more extensively involved in redecision than in the initial decision phase.

Trial-by-trial activity in the regions of the frontoparietal control network in redecision was positively correlated with decision uncertainty level for the initial decision (Fig 3C and S2 Table). Critically, these correlations remained significant even for the correct trials (S1E Fig), indicating that these regions were encoding the decision uncertainty signal rather than the error signal. Furthermore, task difficulty or RT could not explain their association with the decision uncertainty in these regions. The residual fMRI signal changes after the components associated with the task difficulty and RT were regressed out remained highly correlated with decision uncertainty level, but residual fMRI signal changes after the components of the decision uncertainty level were regressed out were not further correlated with the task difficulty and RT. Although the dACC and AIC regions were also partially activated during the initial decision phase (S1A Fig and Fig 3A), this activity—as well as in other regions activated during the same phase—was neither positively nor negatively correlated with the decision uncertainty level (S1D Fig). Activity in the ventromedial PFC (VMPFC) and posterior cingulate cortex (PCC) regions of the default-mode network in redecision were negatively correlated with decision uncertainty level or positively correlated with its inverse—decision confidence (S1F Fig). Thus, the regional activity seen in the frontoparietal control network involved processes intrinsic to redecision but not the activity involved in decision-making for the initial phase.

In the third fMRI experiment (fMRI3, *n* = 25), we confirmed that the strength of activity in the frontoparietal control network depended critically on whether redecision was required after the initial decision phase. When decision uncertainty levels for initial decisions were matched in the two conditions (two-tailed paired *t* test, *t*_25_ = 0.62, *P* = 0.27), activity in the frontoparietal control network was much stronger when redecision was required (‘redecision condition’), in comparison with those when redecision was not required (“non-redecision condition”) (Fig 3D), despite the fact that activation of the frontoparietal control network in the ‘non-redecision condition’ was also significant (S1G Fig) and was correlated with decision uncertainty level as well [25]. Thus, the frontoparietal control network, more strongly activated in redecision, should not only be involved in metacognitive monitoring of decision uncertainty of the initial decision but also in metacognitive control in redecision (Fig 1D). We then putatively defined this frontoparietal control network as the metacognition network.

Because the duration of the redecision phase in fMRI1 was longer (4 s) than that of the initial decision phase (2 s), it raised the question of whether the fMRI activity predominately observed during the redecision phase was induced by the longer exposure, specifically in the trials with more difficult decisions. To address this, we scanned an independent group of participants (fMRI4, *n* = 20) while they underwent the same RDM task as fMRI1 except that the duration of redecision was set to 2 s. The same behavioral and neural results were replicated as in fMRI1 (S4 Fig).

Just as the extent of uncertainty reduction by redecision was found to be highly correlated with the decision uncertainty level of the initial decision (Fig 2F), activity in the regions of the metacognition network were also found to be positively correlated with the extent of uncertainty reduction (S1H Fig). However, the strength of the correlations decreased somewhat after the components associated with the decision uncertainty level were regressed out (S1I Fig). Conversely, correlations with decision uncertainty level in the metacognition network remained significant after the components associated with the extent of uncertainty reduction were regressed out (S1J Fig). These partial correlations complementarily confirmed that the metacognition network in redecision was involved in both metacognitive monitoring and metacognitive control, indicating that the two processes interacted in redecision processing.

### Dissociation of metacognitive monitoring and metacognitive control in redecision within individual participants

**Fig 4:**
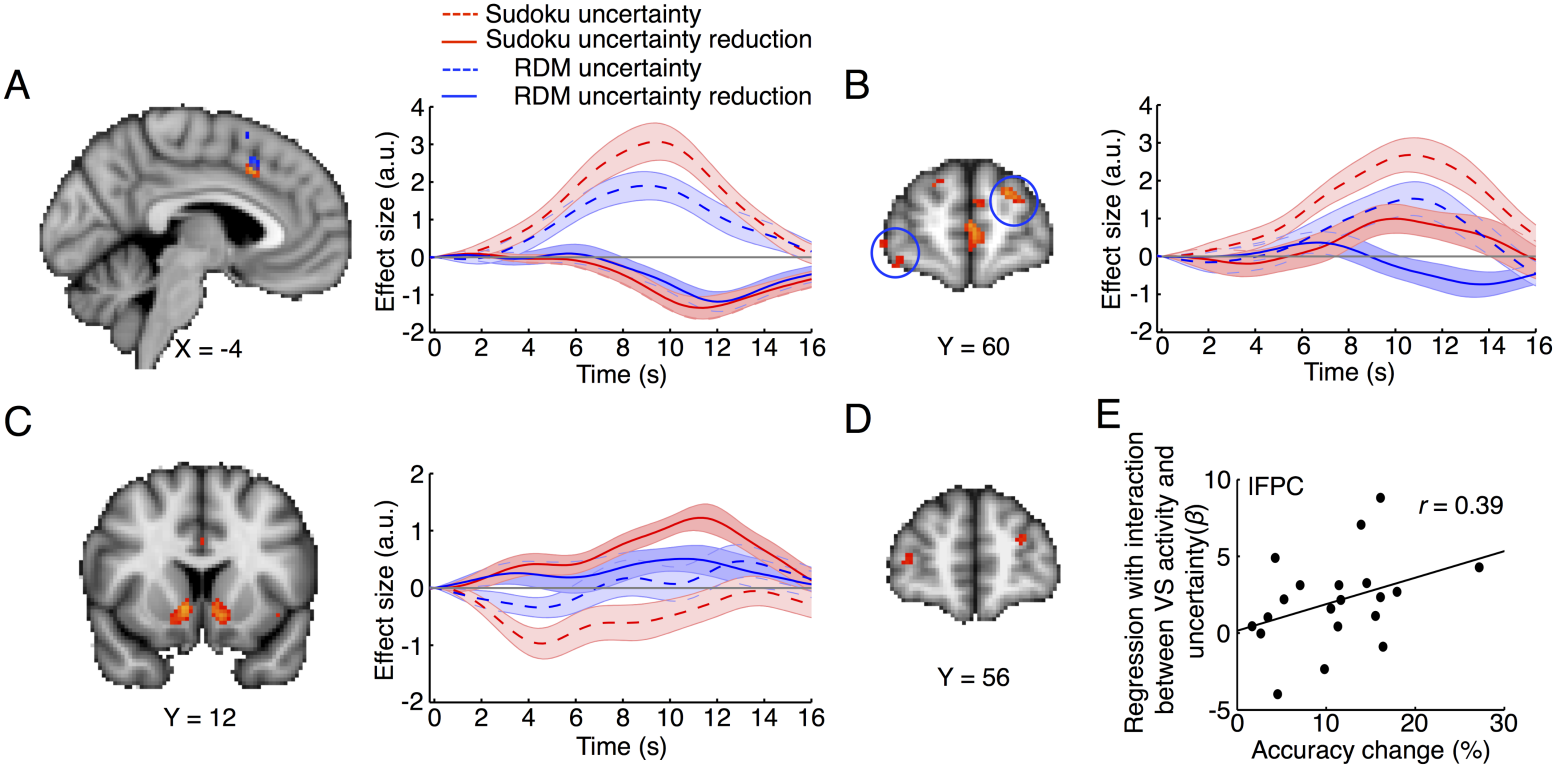
Dissociated metacognitive monitoring in dACC and metacognitive control in the lFPC in redecision processing. (A) dACC activity was negatively correlated with the extent of decision uncertainty reduction after orthogonalization with the decision uncertainty level in both tasks. (B) The lFPC activity was positively correlated with the extent of decision uncertainty reduction in the Sudoku task, but negatively in the RDM task. (C) The VS activity was positively correlated with the extent of decision uncertainty reduction in the Sudoku task, though the early VS activity was negatively correlated with the decision uncertainty level. (D) The lFPC activity was significantly modulated by the VS activity (physiological effect) and the decision uncertainty level (psychological effect) interaction (PPI) in the Sudoku task. (E) Individual accuracy change by redecision was positively correlated with the PPI coupling strength in the lFPC region in the Sudoku task. Time courses are relative to the onset of the initial decision. The broken lines indicate regressions with the decision uncertainty level of the initial decision, and the solid lines indicate regressions with the uncertainty change by redecision (red: the Sudoku task; blue: the RDM task). The data can be found in S1 Data. dACC, dorsal anterior cingulate cortex; lFPC, lateral frontopolar cortex; PPI, psycho–physiological interaction; RDM, random-dot motion; VS, ventral striatum.

The two processes, although interactive, can be dissociated. In the region involved in uncertainty monitoring, activity strength should dynamically represent decision uncertainty level. As decision uncertainty levels were reduced by redecision, the strength of its activity should accordingly be reduced. Therefore, the neural activity change should be negatively correlated with the extent of decision uncertainty reduction. Alternatively, in the region that was critically involved in metacognitive control, its activity should become positively correlated with the extent of decision uncertainty reduction, representing the outcome or the extent of metacognitive control. We found that the activity in the dACC and AIC regions at the late phase of redecision did in fact negatively correlate with the extent of decision uncertainty reduction after the components associated with the decision uncertainty level of the initial decision were regressed out (Fig 4A, S1K and S1L Fig). Conversely, the lFPC activity in the Sudoku task was positively correlated with the extent of decision uncertainty reduction after components associated with the decision uncertainty level were regressed out (Fig 4B), but negatively in the RDM task (Fig 4B and S1I Fig). In addition, VMPFC activity was also positively correlated with the extent of decision uncertainty reduction in both tasks (S1I Fig). The regional activity in the default-mode network appeared intrinsically anticorrelated with the regional activity in the metacognition network (further detail regarding activity in the default-mode network associated with metacognition will be discussed in another study). Thus, the dACC and AIC regions were specifically involved in metacognitive monitoring. In contrast, the lFPC was specifically involved in metacognitive control in redecision, particularly in the Sudoku task. Therefore, their functional roles in metacognition appear to dissociate in redecision processing.

In the Sudoku task, whether the problem would be better solved should be conditioned to individual intrinsic motivation to engage metacognitive control because metacognitive control was effortful. The ventral striatum (VS) activity during the redecision phase was positively correlated with the extent of decision uncertainty reduction in the Sudoku task, but not in the RDM task (Fig 4C). VS might encode the intrinsic motivation or the internal reward on reduction in uncertainty during the redecision phase in the Sudoku task. Critically, the lFPC activity was significantly coupled with the interaction between the VS activity and the decision uncertainty level of the initial decision (Fig 4D; see psycho–physiological interaction [PPI] analysis in Materials and methods). Furthermore, the accuracy change of each participant by redecision was positively correlated with the coupling strength in the Sudoku task (Fig 4E). These results implied that the efficiency of lFPC involvement in metacognitive control in rule-based decision-making tasks (i.e., Sudoku) might be facilitated by the VS activity.

### Dissociation of metacognitive ability in monitoring and control in individual participants

**Fig 5:**
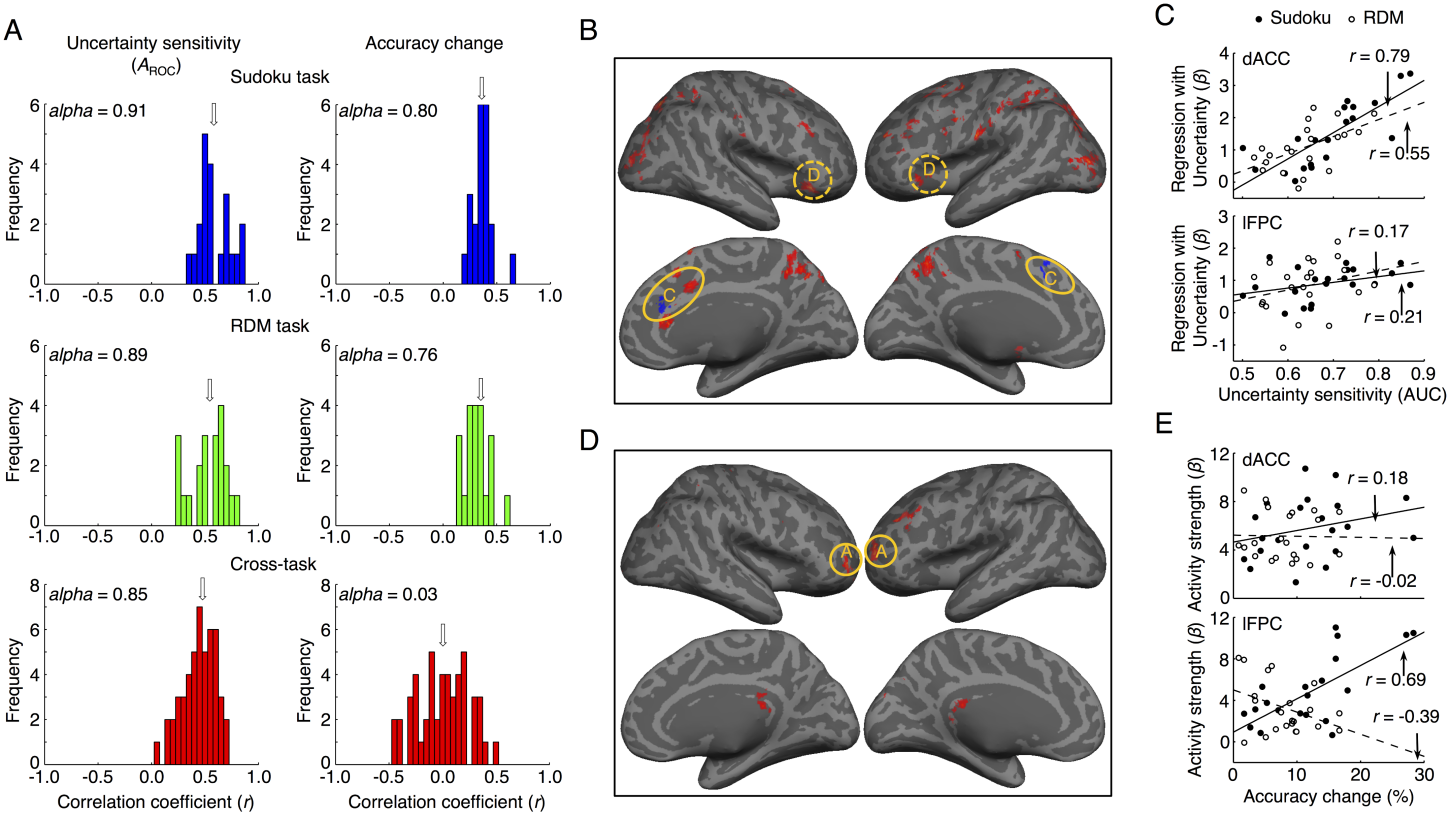
Individual metacognitive uncertainty sensitivity and accuracy change in redecision associated, respectively, with dACC and lFPC activity. (A) The histograms of correlation coefficients of individual uncertainty sensitivity (*A*_ROC_, left column) and individual accuracy change (right column) between different sessions in the Sudoku task and the RDM task as well as across the two tasks. The arrows indicate the medians of the histograms. (B) The individual uncertainty sensitivity (*A*_ROC_) was positively correlated with the uncertainty-level regression *β* values of the fMRI activity mainly in the dACC and AIC regions. (C) The scatter plots of the dACC and lFPC activity regressed with the decision uncertainty level against the individual uncertainty sensitivity. (D) The individual accuracy change was positively correlated with the mean activity predominately in the lFPC region, in the Sudoku task. (E) The scatter plots of the dACC and lFPC mean activity against the individual accuracy change. In panel C and E, the solid lines indicate fitting data in the Sudoku task, and the broken lines indicate fitting data in the RDM task. The conventions in panel B and C are the same as in Fig 3. The data can be found in S1 Data. AIC, anterior insular cortex; dACC, dorsal anterior cingulate cortex; fMRI, functional magnetic resonance imaging; lFPC, lateral frontopolar cortex; RDM, random-dot motion.

The abilities of metacognitive monitoring and control are behaviorally embodied in two components: uncertainty sensitivity and accuracy change, respectively. Throughout all sessions, including fMRI1 and the other repeated behavioral experiments, the individual uncertainty sensitivity was highly consistent across different sessions of the Sudoku task (Cronbach’s *α* = 0.91; Fig 5A, left column, upper panel) and the RDM task (*α* = 0.89, Fig 5A, left column, middle panel), as well as across the two tasks (*α* = 0.85; Fig 5A, left column, lower panel). In contrast, the individual accuracy change in redecision was not consistent across the two tasks (*α* = 0.03; Fig 5A, right column, lower panel), although it was consistent between different sessions of the Sudoku task (*α* = 0.80; Fig 5A, right column, upper panel) and the RDM task (*α* = 0.76; Fig 5A, right column, middle panel). Thus, individual metacognitive abilities of uncertainty monitoring were reliably consistent, but individual metacognitive control was dissociable in the two tasks.

Accordingly, the individual uncertainty sensitivity (*A*_ROC_) was positively correlated with the uncertainty-level regression *β* value of the fMRI signal changes (i.e., neural uncertainty sensitivity), primarily in the dACC and AIC regions (Fig 5B, *P* < 0.001, cluster size = 20; and Fig 5C upper, one-tailed *t* test, Pearson’s *r* = 0.79, *t*_19_ = 5.6, *P* = 6.0 × 10^−6^ in the Sudoku task; *r* = 0.55, *t*_19_ = 2.9, *P* = 0.0049 in the RDM task; S3 Table), but not in the lFPC region (Fig 5B and 5C bottom; Pearson’s *r* = 0.17, *t*_19_ = 0.8, *P* = 0.22 in the Sudoku task; *r* = 0.21, *t*_19_ = 1.0, *P* = 0.17 in the RDM task), commonly in both tasks. The differences of correlations were significant between the two regions (*t*_19_ = 3.8, *P* = 5.6 × 10^−4^ in the Sudoku task; *t*_19_ = 2.3, *P* = 0.016 in the RDM task). In contrast, the individual accuracy change was significantly correlated with the mean activity in the lFPC region (Fig 5D, *P* < 0.001, cluster size = 20; and Fig 5E bottom; Pearson’s *r* = 0.69, *t*_19_ = 4.2, *P* = 2.2 × 10^−4^ in the Sudoku task; *r* = −0.39, *t*_19_ = 1.9, *P* = 0.041 in the RDM task), but not in the dACC region (Fig 5D and 5E upper; Pearson’s *r* = 0.18, *t*_19_ = 0.8, *P* = 0.21 in the Sudoku task; *r* = −0.02, *t*_19_ = 0.09, *P* = 0.47 in the RDM task). When the lFPC activity was stronger, the accuracy change was more in the Sudoku task but became less in the RDM task (Fig 5E). The differences of correlations were significant between the two regions (*t*_19_ = 2.7, *P* = 0.007 in the Sudoku task; *t*_19_ = 1.8, *P* = 0.045 in the RDM task). Thus, the dACC activity (AIC as well) commonly represented individual metacognitive abilities in monitoring of decision uncertainty, whereas the lFPC differentially modulated individual metacognitive abilities in control of decision adjustment—in both the Sudoku and RDM tasks—consistent with their dissociated functional roles in metacognitive monitoring and metacognitive control, respectively.

### Task baseline activity in the metacognitive network predicts individual metacognitive monitoring and control

**Fig 6:**
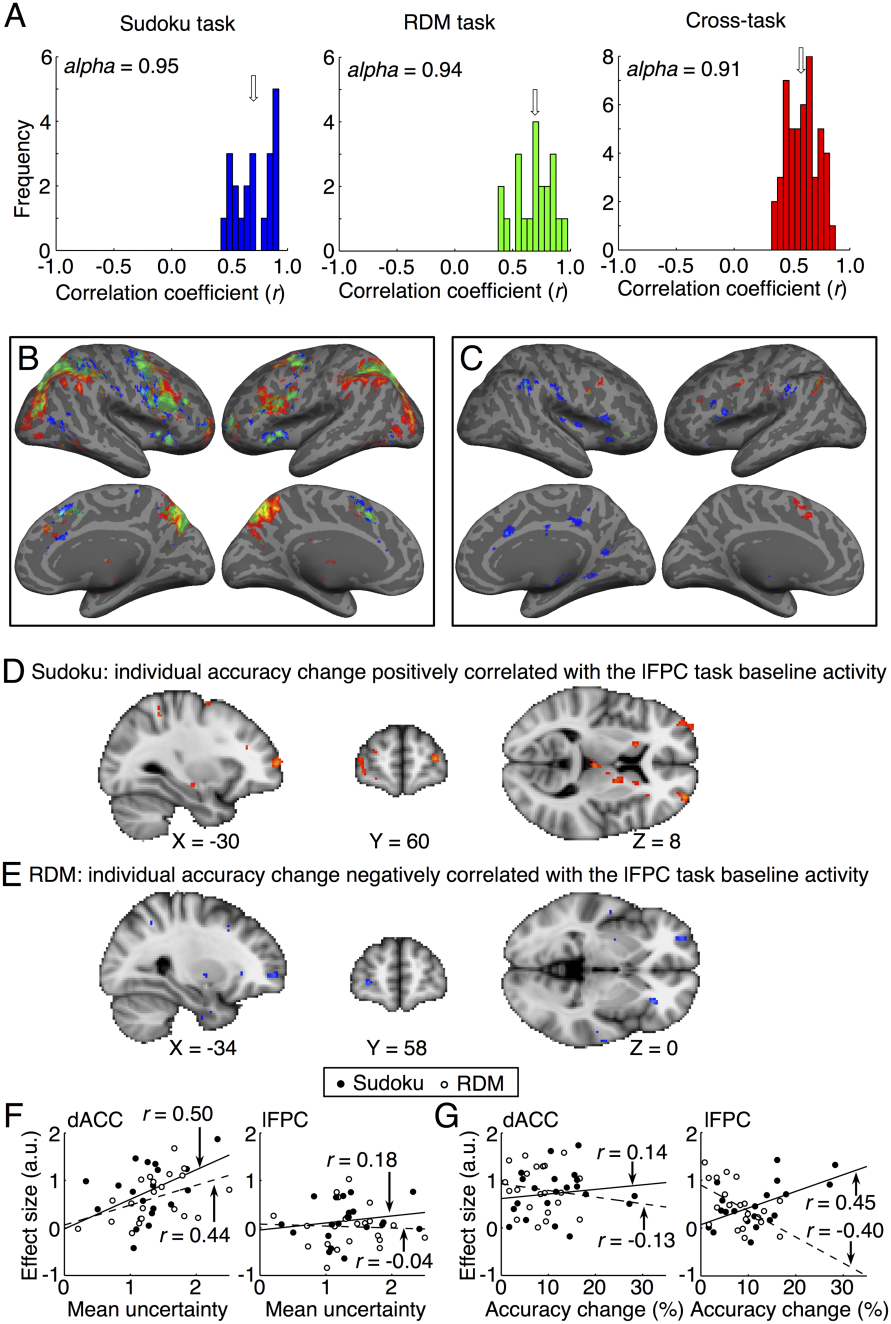
Individual metacognitive abilities predicted by the task baseline activity of the dACC and lFPC regions in the metacognition network in redecision. (A) The histograms of the correlation coefficients of individual uncertainty bias between different sessions of the Sudoku or RDM task as well as across the two tasks. The arrows indicate the medians of the histograms. (B) The task baseline activity (confidence level = 4) in comparison to those of the control trials in the Sudoku task and the RDM task. (C) Positive correlation of task baseline activity during the redecision phase of the task trials with the individual uncertainty bias across the participants. The conventions in panel B and C are the same as in Fig 3. (D) The lFPC task baseline activity was positively correlated with the individual accuracy change across the participants in the Sudoku task. (E) The lFPC task baseline activity was negatively correlated with the individual accuracy change across the participants in the RDM task. (F) The scatter plots of the dACC and lFPC task baseline activity against the individual uncertainty bias. (G) The scatter plots of the dACC and lFPC task baseline activity against the individual accuracy change. In panel F and G, the solid lines indicate fitting data in the Sudoku task, and the broken lines indicate fitting data in the RDM task. The data can be found in S1 Data. dACC, dorsal anterior cingulate cortex; lFPC, lateral frontopolar cortex; RDM, random-dot motion.

The regions of the metacognition network were also activated in the trials of both tasks with confidence level 4 in comparison with their respective control conditions (Fig 6B and S2 Fig). These activity differences might be partially caused by differentially subjective uncertain states of the two conditions that were not reflected by the four-scale confidence ratings (i.e., the ceiling effect). The averaged accuracy was about 80% in the certain trials of the tasks (Fig 2C), but it was about 95% in the control conditions. Nevertheless, the task baseline activity in the certain trials of the tasks could also predict the individual uncertainty monitoring bias and potential abilities of efficient metacognitive control of decision adjustment. Individual uncertainty monitoring bias—as estimated by averaging the decision uncertainty levels of all trials in each session of the tasks, representing the individual’s overconfident or underconfident tendency—was consistent between different sessions in the Sudoku task (*α* = 0.95; Fig 6A, left panel) and in the RDM task (*α* = 0.94, Fig 6A, middle panel), as well as across the two tasks (*α* = 0.91; Fig 6A, right panel). Accordingly, individual uncertainty monitoring bias was positively correlated with the mean task baseline activity in the dACC region (Fig 6C *P* < 0.001, cluster size = 20; and Fig 6F left, Pearson’s *r* = 0.50, *t*_19_ = 2.5, *P* = 0.0096 in the Sudoku task; *r* = 0.44, *t*_19_ = 2.1, *P* = 0.022 in the RDM task) but not in the lFPC region (Fig 6C and 6F right; Pearson’s *r* = 0.18, *t*_19_ = 0.80, *P* = 0.22 in the Sudoku task; *r* = −0.04, *t*_19_ = 0.17, *P* = 0.43 in the RDM task), commonly in both tasks. The differences of correlations were significant between the two regions (*t*_19_ = 2.1, *P* = 0.026 in the Sudoku task; *t*_19_ = 1.8, *P* = 0.042 in the RDM task). Meanwhile, the individual accuracy change in the Sudoku task was positively correlated with the mean task baseline activity in the lFPC region (Fig 6D and 6G right; Pearson’s *r* = 0.45, *t*_19_ = 2.2, *P* = 0.020) but not with that in the dACC region (Fig 6G left; one tailed *t* test, *r* = 0.14, *t*_19_ = 0.62, *P* = 0.27). In contrast, the individual accuracy change in the RDM task was negatively correlated with the mean task baseline activity in the lFPC region (Fig 6E and 6G right; Pearson’s *r* = −0.40, *t*_19_ = 1.9, *P* = 0.035) but not with that in the dACC region (Fig 6G left; *r* = −0.13, *t*_19_ = 0.57, *P* = 0.29). The differences of correlations were significant between the two regions (*t*_19_ = 1.9, *P* = 0.039 in the Sudoku task; *t*_19_ = 1.8, *P* = 0.046 in the RDM task). Furthermore, the differences of correlations with the individual uncertainty monitoring bias and the individual accuracy change in the dACC (*t*_19_ = 2.2, *P* = 0.020 in the Sudoku task; *t*_19_ = 2.8, *P* = 0.0055 in the RDM task), as well as in the lFPC (*t*_19_ = 1.8, *P* = 0.046 in the Sudoku task; *t*_19_ = 2.0, *P* = 0.030 in the RDM task), were significant. Thus, the task baseline activity in the dACC region commonly reflected the individual uncertainty monitoring bias in both tasks, whereas that in the lFPC region could predict the individually differential potential abilities of metacognitive control for decision adjustment in both tasks.

### Subsystems in the metacognition network

**Fig 7:**
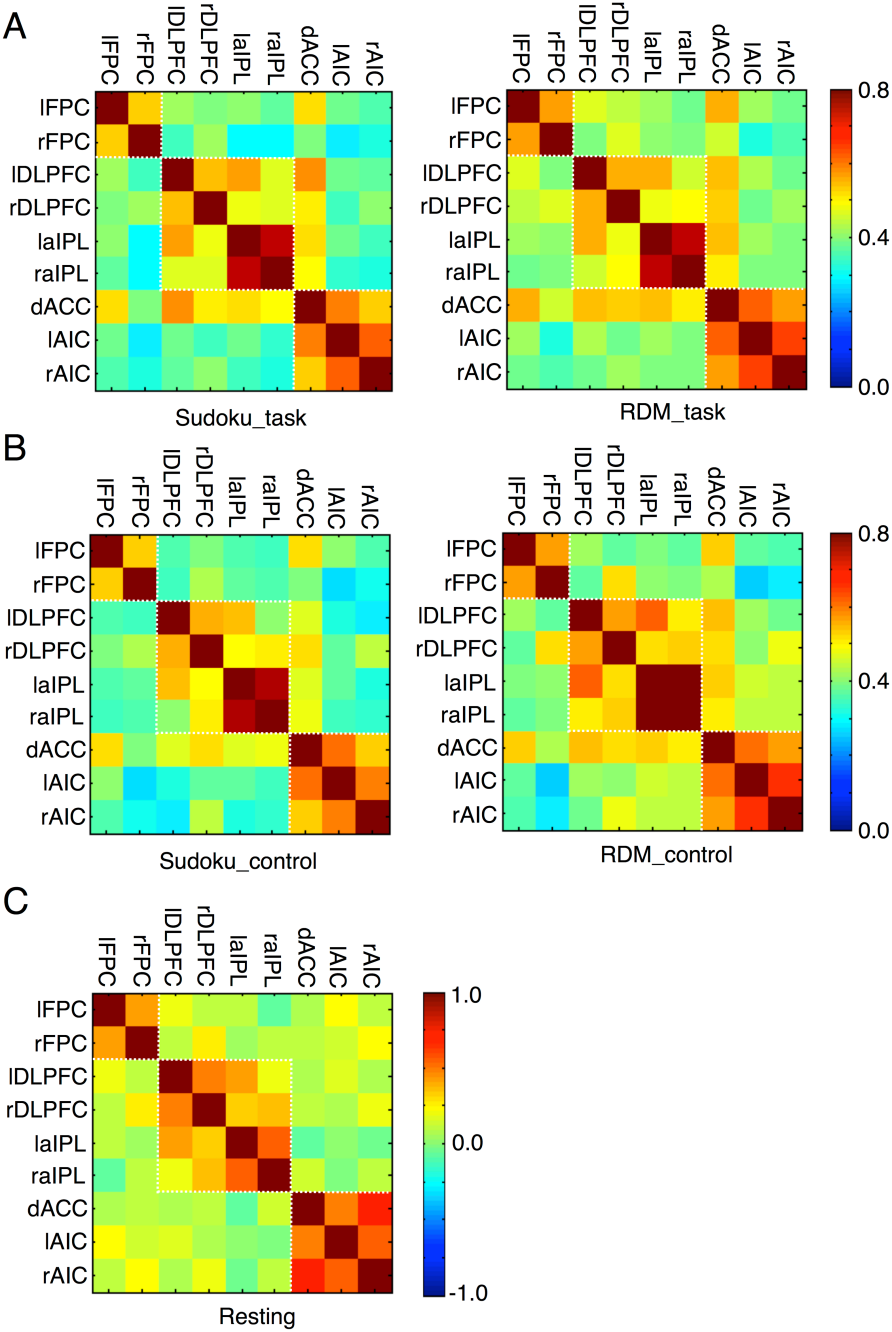
Regional functional connectivity of the metacognition network during (A) task and (B) control conditions in Sudoku and RDM tasks, as well as during (C) resting state. dACC, dorsal anterior cingulate cortex; DLPFC, dorsolateral prefrontal cortex; lAIC, left anterior insular cortex; laIPL, left anterior inferior parietal lobule; lFPC, lateral frontopolar cortex; rAIC, right anterior insular cortex; raIPL, right anterior inferior parietal lobule; RDM, random-dot motion; rFPC, right frontopolar cortex.

Thus far, we have shown that the neural system of metacognition could be dissociated into at least two subsystems: the dACC and AIC regions involved in metacognitive monitoring of decision uncertainty, and the lFPC region involved in metacognitive control of decision adjustment. To further elaborate the subsystems of the metacognition network, we performed analyses of interregional functional connectivity in the metacognition network. By regressing out the mean activity and the modulations by the decision uncertainty level, the RT and the extent of uncertainty reduction, as well as their interactions, we calculated trial-by-trial correlations between each pair of regions in the metacognition network (see Materials and methods). The interregional functional connectivity patterns in both the task condition (Fig 7A) and the control condition (Fig 7B) were almost identical across the two types of tasks and were also similar to those at the resting state (Fig 7C). The interregional functional connectivity patterns consistently showed that the metacognition network might be divided into three subsystems: the lFPC region; the dACC and AIC regions; and the DLPFC and aIPL regions. The interregional functional connectivity within each of the subsystems was systematically stronger than that across the subsystems (paired *t* test, *P* < 0.05 in all comparisons). So far, the functional roles of the subsystem consisting of the DLPFC and aIPL regions in metacognition remain unclear. It is worth noting that the functional connectivity between the dACC and the regions of the other two subsystems in the task conditions was numerically stronger than the corresponding one at the resting state but was not statistically significant.

## Discussion

In the present study, we utilized a novel decision–redecision paradigm to examine the behavioral and neural associations of metacognitive processing in redecision, as compared to the processing in an initial decision. The robust findings from our study showed that individual uncertainty sensitivity (both *A*_ROC_ and *r*_RT-uncertainty_) remained markedly stable over two consecutive decisions on the same situational task, between different sessions of the same tasks, and across the different tasks. This indicates that individual uncertainty sensitivity was independent of evidence accumulation or the form of the decision-making process. These findings provide evidence to contradict the theoretical prediction of Theory 2. If the processes of metacognitive processing and decision-making were integrated in one network, it should follow that, as more evidence is accumulated after redecision, the uncertainty sensitivity (i.e., *A*_ROC_) should be also improved [28]. Our study did not support that but rather led us to suggest the existence of an additional neural process in the brain to nonuniformly transform evidence accumulated in the decision-making processes to neural signals encoding decision confidence/uncertainty. The decision confidence/uncertainty should be much constrained by this neural process, as proposed by Theory 1. Using fMRI, we identified patterns of neural activity in the frontoparietal control network that were more extensively involved in redecision than with initial decisions. Furthermore, activity in the regions of this network was positively scaled with decision uncertainty and became stronger in the condition requiring redecision than that in the condition not requiring redecision after the initial decision. These findings suggest that this network is involved both in metacognitive monitoring of decision uncertainty and metacognitive control of decision adjustment. Taken together, the evidence supports the theoretical proposal that metacognition utilizes a separate neural system to monitor and control decision-making (i.e., Theory 1). We have putatively referred to the network revealed by our experiments as the metacognition network. We further propose that this network could be segregated into three subsystems (as shown in Fig 7).

The subsystem consisting of the dACC and AIC regions was involved in metacognitive monitoring of decision uncertainty, common in the two tasks. The neural uncertainty sensitivity (the uncertainty-level regression *β* value) in the two regions was highly correlated with the behavioral uncertainty sensitivity. Furthermore, their task baseline activity could predict the individual uncertainty bias. Thus, the decision uncertainty signal could be finally represented by the dACC and AIC activity, which might be the outcome of transforming the uncertainty information from the decision-making process [29]. We thus inferred that uncertainty monitoring might indeed consist of two-order processes. We suggest the possibility that the first-order process coincides with the decision-making process that simultaneously generates the uncertainty information, implicitly associated with decision uncertainty, as proposed by Theory 2, and that the second-order process then transforms this uncertainty information from different decision-making processes into common decision uncertainty scales, which are encoded in the dACC and AIC regions, as activity observed in this study would support. This hypothesis then integrates the two theories together and consistently accounts for observed evidence from both sides. It is worth noting that our results differed from previous neuroanatomical studies showing that the lFPC region was associated with individual behavioral uncertainty sensitivity [9, 18].

The dACC and AIC regions have been well recognized for their involvement in conflict and error monitoring of the preceding cognitive processes to signal the need for more control [30–32]. Our results suggest that it is decision uncertainty, rather than decision error or conflict information, that serves as the primary signal to evoke monitoring [25]. Our findings are also profoundly different from previously reported accounts of dACC function in performance monitoring. First, there was no explicit feedback or cue to indicate whether the decision was correct or incorrect. The participants evaluated decision uncertainty via individual internal signals rather than external cues. Secondly, the task difficulty and RT does not explain the dACC and AIC activity in association with decision uncertainty. The dACC and AIC regions have been shown to broadly monitor subjective feelings such as pain, emotion, and others [33]. Critically, the salient information that elicits conscious monitoring in these regions is not necessarily from the somatosensory stimulation [34]. Similarly, the prospective monitoring of uncertainty in judgments of learning (JOL) and feeling-of-knowing (FOK) has also been shown to activate these regions, prior to execution of the decision-making tasks [35]. Therefore, decision uncertainty monitoring in the dACC and AIC regions should be domain general, independent of the sources of uncertainty information and the forms of decision-making tasks. In short, the individual uncertainty sensitivity is a unique and core trait of each individual decision maker, presumably dependent on the circuit of the dACC and AIC regions [33].

Decision uncertainty monitoring could be a bottom-up process. It occurred automatically without any explicit requirement of redecision (fMRI3, S1G Fig) [25]. However, the subsequent metacognitive control of decision adjustment should require top-down cognitive control. In the Sudoku task, lFPC activity was positively correlated with the extent of decision uncertainty reduction within individual participants and the accuracy change by redecision across the participants, suggesting critical involvement of the lFPC region in metacognitive control. Uncertainty-driven exploration could be a critical process in metacognitive control [25, 36–39]. Revising foregone decisions usually requires an exploration of alternative-solution approaches because the previously used solution approach would likely lead to the same unsatisfactory solution. Through exploration of alternative solutions, a more satisfactory option could be found by which the decision uncertainty would therefore be reduced. During this process of exploration, strategy management could be a key function of lFPC involvement in metacognitive control. This top-down strategic signal might regulate the activity in other frontal cortical areas and posterior parietal cortex, to execute the processes of altering the previous uncertain choice [25, 36, 39] or to explore a non-default option [37, 38].

This would lead to the expectation that the lFPC would not be involved in metacognitive control in the RDM task because revising the preceding perceptual decision might simply require more attention to the stimulus in redecision to continue evidence accumulation, not necessarily exploration. However, lFPC activity remained activated as well and was negatively correlated with the extent of decision uncertainty reduction and accuracy change. It is possible that the process of exploration in the lFPC might be competitive with the simultaneous process of exploitation in the posterior brain areas when these two-level systems are not well coordinated [40, 41]. Indeed, an FPC lesion in nonhuman primates enhanced the animals’ performance of a well-learned decision-making task [42]. However, it remains enigmatic why the lFPC was kept activated when it was not necessary and would not facilitate the engaging task. Presumably, the signal for increased control derived from the dACC region that is sensitive to decision uncertainty might nonselectively activate the lFPC because lFPC activity was also conditioned by decision uncertainty. The automaticity of eliciting lFPC involvement in metacognitive control may facilitate uncertainty resolution in the majority of difficult real-world situations, to relieve effort for engagement in metacognitive control, but failure of disengagement could impair the performance adjustment in simple tasks. Instead, intrinsic motivation might boost metacognitive control of decision adjustment in demanding tasks through VS activity.

Metacognitive control is a form of cognitive control; however, not all forms of cognitive control are metacognitive. Although the Sudoku task and the RDM task appeared very different, to our surprise, the fMRI activation patterns associated with the decision-making process in the initial decisions were quite similar between the two tasks. Critically, the IFJ at the posterior PFC was commonly activated. IFJ is ubiquitously engaged in online task execution, in involving cognitive control [43, 44], and attention [45]. Thus, IFJ might play a critical role in object-level cognitive control, generally in decision-making tasks [25, 46, 47]. The segregation of the meta-level cognitive control in the anterior PFC and the object-level cognitive control in the posterior PFC is aligned with the hypothesis of the rostrocaudal functional division of the PFC in cognitive control [25, 48, 49]. However, the PFC functional division proposed in the current paper is subject to the strategy of task implementation [25] rather than to level of task complexity [48, 49]. The initial decision-making merely recruits the posterior PFC to implement the default strategy of exploiting routine processes, whereas metacognition is evoked when the initial decisions are uncertain, recruiting the frontoparietal control network, including the anterior PFC, to control exploration of alternative processes [50]. Therefore, the metacognition process in redecision is not incorporating prior information acquired in the initial decision, but rather it is prone to altering the initial decision.

There were some potential pitfalls for the fMRI data analyses in the current study. Because the metacognition process should automatically accompany the decision-making process with uncertainty, it excludes the possibility of inserting time jitters between the initial decision phase and the redecision phase, as conventionally used in fMRI paradigms. Thus, the two events of the decision-making process and the metacognition process in the general linear models (GLMs) could be collinear and result in inflation of standard errors of the estimated parameters for the regions involved in both processes. Fortunately, the activation of the regions of interest (ROIs) predominately involved in metacognition appeared in the redecision phase. Of note, the variance inflation factor (VIF) was approximately 2.4, which suggests that the collinearity of the GLMs used in the current study was not severe.

**Fig 8:**
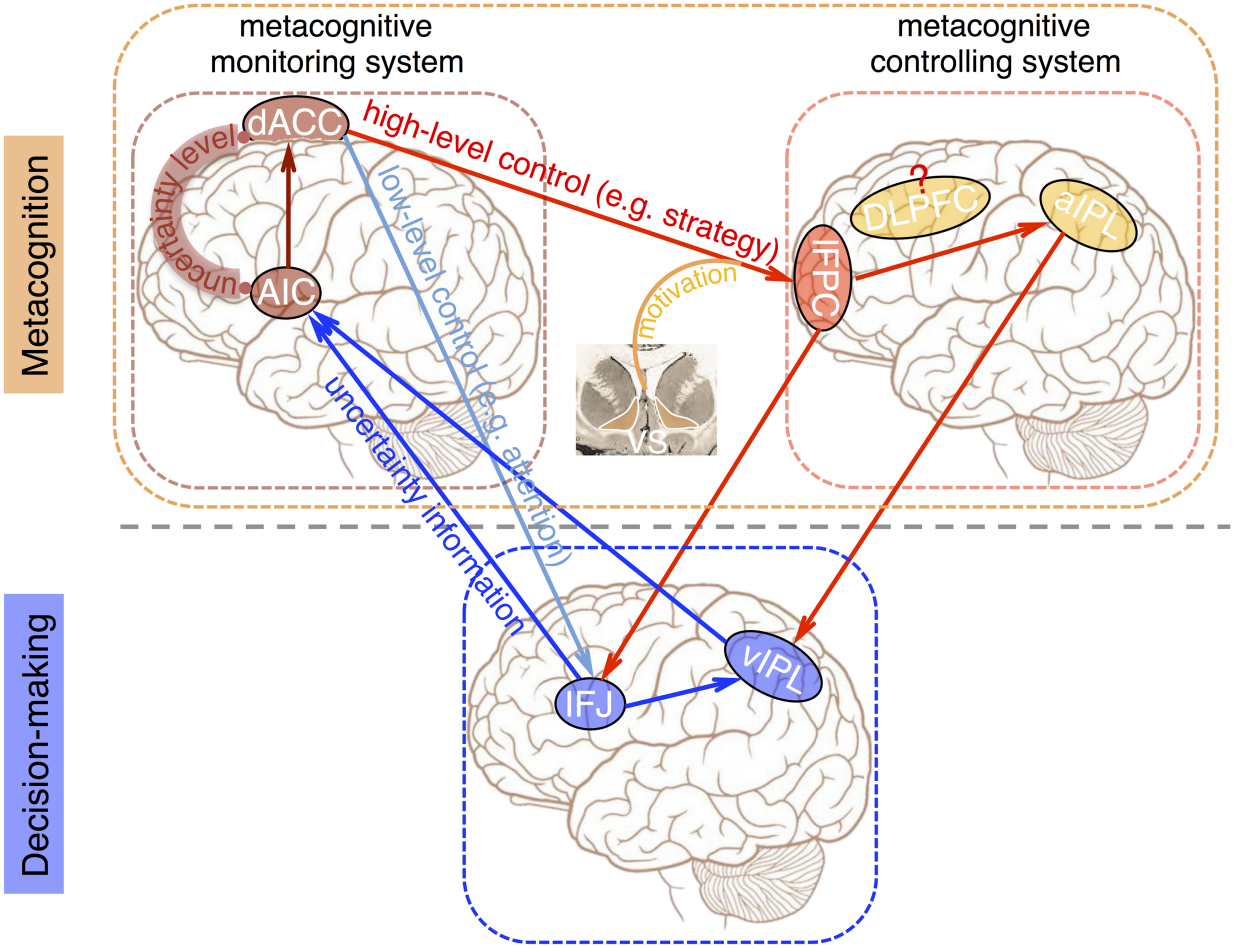
The functional architecture of the metacognition neural system. The scheme of the functional architecture of the metacognition neural system and its interactions with the decision-making neural system, as synthesized from the converging results in the current study. The metacognition neural system is composed of the metacognitive monitoring system (dACC and AIC) and the metacognitive control system (lFPC). The decision-making neural system and the metacognition neural system form a closed-loop system. AIC, anterior insular cortex; aIPL, anterior inferior parietal lobule; dACC, dorsal anterior cingulate cortex; DLPFC, dorsolateral prefrontal cortex; IFJ, inferior frontal junction; lFPC, lateral frontopolar cortex; vIPL, ventral inferior parietal lobule.

In summary, we have constructed and proposed the extent and generality of the functional architecture of the metacognition neural system, which is separate from the decision-making neural system (Fig 8). The metacognition neural system is composed of the metacognitive monitoring system and the metacognitive control system. The metacognitive monitoring system, consisting of the dACC and AIC regions, is domain general. It reads out the uncertainty information from the decision-making process and quantitatively encodes the decision uncertainty states. The metacognitive control system of the lFPC region implements high-level cognitive control (e.g., strategy), dominant in rule-based and abstract inference tasks (e.g., the Sudoku task), and may compete with low-level cognitive control (e.g., attention), dominant in perceptual tasks (e.g., the RDM task). The high-level cognitive control by the lFPC region could be modulated by intrinsic motivational signals from the VS region. These two subsystems separately monitor and control the decision-making system, in which the IFJ region is critically involved. Thus, the decision-making neural system and the metacognition neural system form a closed-loop system to control and adapt our behavior towards desired goals.

## Materials and methods

### Ethics statement

All participants were university students, who were recruited through a campus bulletin board system (BBS). Informed consent was obtained from each individual participant in accordance with a protocol approved by Beijing Normal University Research Ethics Committee.

### Participants

Twenty-one participants (19–33 y old, 12 female) took part in the main fMRI experiment (fMRI1) and the resting fMRI experiment. Out of them, 16 participants (19–33 y old, 9 female) took part in all sessions of the repeated behavioral experiments. In addition, 17 participants (19–25 y old, 10 female) took part in the second fMRI experiment (fMRI2), 25 participants (19–27 y old, 14 female) took part in the third fMRI experiment (fMRI3), and 20 participants (19–26 y old, 11 female) took part in the fourth fMRI experiment (fMRI4).

### RDM task

In an aperture with a radius of 3 degrees (visual angle), about 300 white dots (radius: 0.08 degrees; density: 2.0%) on a black background that were moving in different directions at a speed of 8.0 degrees/s were displayed. The time span of the movement of each dot lasted for 3 frames. A portion of dots was moving in the same direction (one of the four directions: left, right, up, and down), while the others were moving in different, random directions. The participant was required to discriminate the net motion direction. According to the proportion of coherently moving dots, discrimination difficulty was classified into 10 levels (Fig 2B), for which the movement coherence varied from 1.6% to 51.2%; coherence of moving dots in the control condition was 100%.

### Sudoku task

To fill a 4 × 4 grid matrix, each digital number from 1 to 4 should be filled in once and only once in each column, each row, and each corner with 4 grids. The task used in the present study required participants to fill in a target grid with a digital number from 1 to 4 in a partially completed Sudoku problem. Each problem had a unique solution. A Sudoku generator (custom codes) was used to create thousands of different Sudoku problems. Problem difficulty was classified into 10 levels according to the minimum number of logic operation steps required to arrive at the solution; this classification scheme largely matched with participants’ reported subjective difficulty level (Fig 2B). In the control condition, the presented problem was made up of symbols (“#”) replacing the digital numbers except for the space in the target grid where the digital number was illustrated. Thus, the participant only needed to press the corresponding button.

### Learning procedure

Participants were trained in the cognitive skills used to solve the 4 × 4 Sudoku problems under experimenters’ guidance for at least two h per d over a continuous span of 4 d. The participants practiced solving problems with no time constraints first in two to four runs of 40 problems at a set difficulty level. Once the average accuracy of a session crossed 90%, s/he then practiced solving problems at the same level within 2 s. Once the average accuracy of the run in a 2-s time frame reached 70%, the participant repeated these steps at the next level of difficulty. After the 4-d intensive training program, each participant had attained high-level proficiency in solving 4 × 4 Sudoku problems in 2 s, as the mean task difficulty finally approached the fifth level.

### Task sequences

The sequences of both the Sudoku and RDM tasks were identical. In fMRI1, each trial started with a green-cross cue to indicate that the task stimulus would be presented 1 s later. The stimulus was presented for 2 s, then four options were presented, and the participant made a choice within 2 s. After a choice was made, four confidence level ratings from 1 (lowest) to 4 (highest) were presented, and the participant reported their confidence rating within 2 s. The same stimulus was immediately presented again for 4 s, and the participant again selected a choice and again reported their confidence rating. Each trial lasted for 15 s. The control trials were intermingled with task trials. The sequence of the control trials was identical to that of the task trials. In each task, there were 4 runs, and each run consisted of 30 task trials and 10 control trials. The task difficulty of each trial was adjusted by a staircase procedure through which one level was upgraded after two consecutive correct trials, was downgraded by one level after two consecutive erroneous trials, and was kept at the same level otherwise, so that the mean accuracy converged at about 50%. Prior to each experiment, two runs were carried out to allow each participant to practice and stabilize performance. The Sudoku problems used in the learning and practice sessions were different from those used in the fMRI and behavioral experiments. In addition, a 10-min resting fMRI experiment was conducted when the participant was in a resting state with eyes opened.

The second fMRI experiment (fMRI2, Fig 4 and S1C Fig) was carried out to examine whether the metacognition network would also be essentially involved in the decision-making process in the initial decision if a new Sudoku problem or new RDM stimulus was presented during the redecision phase, following a control stimulus presentation in the initial decision phase, as used in fMRI1. In fMRI2, a randomized selection of a control stimulus, a new Sudoku problem, or a new RDM stimulus was presented in the redecision phase. The appearance of a new stimulus in the redecision phase occurred in half of the trials. The Sudoku problems or RDM stimuli used in this experiment were selected from those at the middle level of task difficulty. This design was used to reduce the participant’s decision uncertainty in this experiment by controlling the difficulty of the task. In all other ways, the task sequence was the same as that used in fMRI1. In total, there were 120 trials across two runs in each task.

The third fMRI experiment (fMRI3, Fig 3D and S1E Fig) was carried out to compare brain activity in a redecision-task condition (like the task in fMRI1) to activity in a task that did not require redecision. In non-redecision trials, a control stimulus was presented in the redecision phase, therefore no redecision was required. The task sequence was the same as the design used in fMRI1 with the exception that the presentation time of the stimulus was 3 s during the redecision phase. In each task, the ‘redecision’ condition and the ‘non-redecision’ condition were randomized, and each consisted of 60 trials across 3 runs.

The fourth fMRI experiment (fMRI4, S4 Fig) was carried out to confirm that the engagement of metacognition was independent of the duration of redecision. The task sequence was exactly the same as was used in fMRI1, with the exception that the redecision phase was 2 s instead of 4 s. Only the RDM task was used in this experiment. In total, there were 120 task trials and 40 control trials across 4 runs.

### Stimuli presentation

In the fMRI experiments, the participants viewed images of the stimuli on a rear-projection screen through a mirror (resolution, 1,024 × 768 pixels; refresh rate, 60 Hz). Normal or corrected-to-normal vision was achieved for each participant. All images were restricted to 3 degrees surrounding the fixation cross.

### fMRI experiments

All fMRI experiments were conducted using a 3 T Siemens Trio MRI system with a 12-channel head coil (Siemens, Germany) after the 4-d Sudoku training. Functional images were acquired with a single-shot gradient echo T_2_* echo-planar imaging (EPI) sequence with volume repetition time (TR) of 2 s, echo time (TE) of 30 ms, slice thickness of 3.0 mm, and in-plane resolution of 3.0 × 3.0 mm^2^ (field of view [FOV]: 19.2 × 19.2 cm^2^; flip angle [FA]: 90 degrees). Thirty-eight axial slices were taken, with interleaved acquisition, parallel to the anterior commissure–posterior commissure (AC–PC) line.

### Behavioral experiments

To test the reliability of the participants’ metacognitive abilities, behavioral experiments were carried out using the same paradigms as the Sudoku and RDM tasks. Each of the participants completed 6 sessions of behavioral experiments on consecutive days. Each session was composed of 4 runs of the Sudoku task and 4 runs of the RDM task, the same as was used in fMRI1.

### Behavioral data analyses

A nonparametric approach was employed to assess each participant’s uncertainty sensitivity. The ROC curve was constructed by characterizing the incorrect probabilities with different uncertainty levels for initial decisions as thresholds. The area under curve (AUC) was calculated to represent how well the participant was at detecting and rating their decision uncertainty [9]. The individual uncertainty bias was estimated by the mean uncertainty level of each session, regressed out the factor of *A*_roc_. The accuracy change was the change in mean accuracy from the first decision to the second decision. The individual uncertainty sensitivity and uncertainty bias, as well as accuracy change, were calculated for each session of the fMRI and behavioral experiments.

### fMRI analyses

The analysis was conducted with FMRIB’s Software Library (FSL) [51]. To correct for the rigid head motion, all EPI images were realigned to the first volume of the first scan. Data sets in which the translation motions were larger than 2.0 mm or the rotation motions were larger than 1.0 degree were discarded. It turned out that no data had to be discarded in the fMRI experiments. The EPI images were first aligned to individual high-resolution structural images and were then transformed to the Montreal Neurological Institute space by using affine registration with 6 degrees of freedom and resampling the data with a resolution of 2 × 2 × 2 mm^3^. A spatial smoothing with a 4-mm Gaussian kernel (full-width at half-maximum) and a high-pass temporal filtering with a cutoff of 0.005 Hz were applied to all fMRI data.

Each trial in fMRI1 was modeled with three regressors. The first regressor represented the decision-making process in the initial decision, which was time-locked to the onset of the first stimuli presentation, with summation of the presentation time (2 s) and the differential RT from the mean RT of control trials as the event duration. The second regressor represented the neural process following the initial decision, including the metacognition process and the decision-making process in the redecision, and was time-locked to the onset of the first confidence judgment—with summation of the confidence report, the second presentation time (4 s) of the stimuli, and the differential RT from the mean RT of control trials as the event duration. The third regressor represented the baseline during the intertrial intervals (ITIs), time-locked to the onset of ITI, with the ITI duration as the event duration. The uncertainty level of the initial decision, the RT, and the level of uncertainty reduction (differences in the uncertainty level between the final decision and the initial decision) were implemented as modulators of the second regressor by demeaning the variances of the uncertainty level (Fig 3C) and consequently orthogonalizing the RT and the level of uncertainty reduction with each other (Fig 3A–3C and S1I Fig), or reversing the orthogonalization order (S1J Fig). It should be noted that the orthogonalization processes were equal to stepwise regression analyses on these covariates. The same analyses were applied to the fMRI data of fMRI2 and fMRI3.

For group-level analysis, we used FMRIB’s local analysis of mixed effects (FLAME), which model both “fixed effects” of within-participant variance and ‘random effects’ of between-participant variance using Gaussian random-field theory. Statistical parametric maps were generated by a threshold with *P* < 0.05 with false discovery rate (FDR) correction, unless noted otherwise. The regressions of the individual uncertainty sensitivity (*A*_ROC_), the individual RT–uncertainty correlation coefficient, the individual mean uncertainty level, and the individual accuracy change with the *β* weights of uncertainty levels (Figs 5B, 5D and 6C)—or with the task baseline activity (Fig 6C–6E)—were calculated at the third level of group analyses. For these analyses, statistical parametric maps were generated by a threshold of *P* < 0.001 with the cluster-size threshold as 20 (family-wise error correction).

### ROI analyses

The ROIs of the metacognition network were defined by the voxels that were significantly activated during the redecision phase in the task trials compared to those during the same phase in the control trials across both tasks using conjunction analysis (*P* < 0.001, cluster-wise correction; green areas in statistical parametric maps). ROI analyses were obtained from both hemispheres of the same region. The VS ROI was anatomically defined by the striatum atlas of FSL templates [52]. The time courses were derived from the ROIs, calculating a mean time course within an ROI in each participant individually. We then averaged the time courses of the same condition across the participants (S2 and S3 Figs); or, we oversampled the time course by 10 and created epochs from the beginning of an event onward, then applied the corresponding GLM to every pseudo-sampled time point separately. By averaging the *β* weights across participants, we created the time courses shown in Fig 4. SEMs were calculated between participants.

### PPI analysis

The PPI analysis (Fig 4D) was conducted with the demeaned VS time courses after removing the mean activity and the component correlated with the uncertainty level as the physiological factor, and the uncertainty level convolved with the canonical hemodynamic response function (HRF) during the redecision phase as the psychological factor. The two factors per se, and the interaction between the two factors as confound regressors, were put together into a new GLM analysis across the whole brain.

### Functional connectivity analyses

Functional connectivity analyses were independently conducted for the task and resting fMRI data. For the task fMRI data in each ROI, the mean activity and the components associated with the uncertainty level, RT, level of uncertainty reduction, and their interactions were regressed out; the residual time courses were then averaged across the voxels of the region and segmented into the individual trials of the task and control conditions in the Sudoku and RDM task, respectively. The segmented data of each trial were then modeled using a single regressor during the redecision phase convolved with the canonical HRF, and then a regression value was obtained for each trial. The correlation coefficient of the regression values between each pair of the ROIs in the metacognition network was calculated across the trials of the task or control condition in each participant. Finally, the averaged correlation coefficients were shown (Fig 7A and 7B). For the resting fMRI data, the standard processing was carried out [53], and the averaged correlation coefficients were shown (Fig 7C).

## Supporting information

S1 Data Excel spreadsheet containing, in separate sheets, the underlying numerical data and statistical analysis for Figure panels 2B, 2C, 2D, 2E, 2F, 2G, 2H, 2I, 4A, 4B, 4C, 4E, 5A, 5C, 5E, 6A, 6F, 6G, 7A, 7B, 7C, S2, S3, S4A, S4B, S4C, and S4E. (XLSX)

S1 Table Activations between the task and control conditions. (DOCX)

S2 Table Activations correlated with the uncertainty level and the uncertainty reduction during the redecision phase. (DOCX)

S3 Table Activations positively correlated with the individual uncertainty sensitivity and the individual accuracy change. (DOCX)

S1 Fig Collective statistical parametric maps in the experiments. (A) Activations during the decision phase compared with those during the ITI period in fMRI1. (B) Activations during the redecision phase compared with those during the decision phase in fMRI1. (C) Activations of the initial decision during the redecision phase compared with those of the control condition during the same phase in fMRI2. (D) Positive correlation of activity during the decision phase with the decision uncertainty level in fMRI1 (there were also no negative correlations). (E) Positive correlation of activity during the redecision phase of the correct trials with the decision uncertainty level of the initial decision in fMRI1. (F) Negative correlation of activity during the redecision phase with the decision uncertainty level of the initial decision in fMRI1. (G) Activations during the redecision phase without requirement to decide the previous situation again (‘non-redecision’ condition) compared with those of the control trials during the same phase in fMRI3. (H) Positive correlation of activity during the redecision phase with the level of decision uncertainty reduction in fMRI1. (I) Positive correlation of activity during the redecision phase with the level of decision uncertainty reduction after orthogonalization with the decision uncertainty level in fMRI1. (J) Positive correlation of activity during the redecision phase with the decision uncertainty level after orthogonalization with the level of decision uncertainty reduction in fMRI1. (K) Negative correlation of activity during the redecision phase with the level of decision uncertainty reduction after orthogonalization with the decision uncertainty level in fMRI1. (L) Positive correlation of activity during the redecision phase with the interaction between the decision uncertainty level and the level of decision uncertainty reduction in fMRI1. The conventions are the same as in Fig 3. fMRI, functional magnetic resonance imaging; ITI, intertrial interval.
(TIF)

S2 Fig The time course of fMRI signal changes in regions of the metacognition network at different confidence levels in fMRI1. The time zero was the onset of the initial decision. The blue shadow represents the initial decision-making period, and the yellow shadow represents the redecision period. The data can be found in S1 Data. AIC, anterior insular cortex; aIPL, anterior inferior parietal lobule; dACC, dorsal anterior cingulate cortex; fMRI, functional magnetic resonance imaging; lFPC, lateral frontopolar cortex; mDLPFC, middle dorsolateral prefrontal cortex.
(TIF)

S3 Fig The time courses of the fMRI signal changes in the dACC, mDLPFC, and lFPC regions when the initial decision was conducted during the second phase in fMRI2. The time zero was the onset of the stimulus presentation in the second phase. The participant made the initial decision in the second phase, and the decision duration lasted for 4 s, longer than the initial decision period (2 s) in fMRI1. It should be noted that there was no significant activity in the mDLPFC and lFPC in the task trials, whereas the weak dACC activity in the task trials was delayed for over 3 s from the onset of the stimulus presentation. The data can be found in S1 Data. dACC, dorsal anterior cingulate cortex; fMRI, functional magnetic resonance imaging; lFPC, lateral frontopolar cortex; mDLPFC, middle dorsolateral PFC.
(TIF)

S4 Fig The behavioral and fMRI results in fMRI4. (A) The individual uncertainty sensitivity (*A*_ROC_, blue circles) and decision accuracy (red diamonds) in the initial decision. (B) The relationship between the extent of decision uncertainty reduction and the accuracy change by redecision. (C) The individual uncertainty sensitivity (*A*_ROC_) in the initial and final decisions. There was no difference between the two uncertainty sensitivities (*t*_19_ = 1.3, *P* = 0.10). (D) The *z*-statistic activation map of the task trials in comparison with those of the control trials during the redecision phase. *z* = 3.1, *P* < 0.05, FDR correction. (E) The dACC and FPC activity was positively correlated with the decision uncertainty level and negatively correlated with the extent of decision uncertainty reduction. The data can be found in S1 Data. dACC, dorsal anterior cingulate cortex; FDR, false discovery rate; fMRI, functional magnetic resonance imaging; FPC, frontopolar cortex.
(TIF)

## Abbreviations

AIC: anterior insular cortex
aIPL: anterior inferior parietal lobule
AUC: area under curve
dACC: dorsal anterior cingulate cortex
EPA: echo-planar imaging
FA: flip angle
FDR: false discovery rate
fMRI: functional magnetic resonance imaging
FOK: feeling-of-knowing
FOV: field of view
GLM: general linear model
HRF: hemodynamic response function
IFJ: inferior frontal junction
ITI: intertrial interval
JOL: judgments of learning
lFPC: lateral frontopolar cortex
mDLPFC: middle dorsolateral PFC
PCC: posterior cingulate cortex
PFC: prefrontal cortex
PPI: psycho–physiological interaction
RDM: random-dot motion
ROC: receiver operating characteristic
ROI: region of interest
RT: response time
VIF: variance inflation factor
VMPFC: ventromedial PFC
VS: ventral striatum
