# Supplementary material for: The neural system of metacognition accompanying decision-making in the prefrontal cortex
Source: PLoS Biol. 2018 Apr 23;16(4):e2004037. doi: 10.1371/journal.pbio.2004037 (PMC5933819; doi:10.1371/journal.pbio.2004037)
Supplement: S1 Table — (DOCX) [file pbio.2004037.s002.docx]

S1 Table. Activations between the task and control conditions.

| **Task** | **Anatomical Region** | **Hemispheres** | **Coordinate**  **(x, y, z)** | **Maximum**  **Z value** |
| --- | --- | --- | --- | --- |
| **Task – control during the decision phase** | | | | |
| Sudoku | inferior frontal junction (IFJ) | L | -46, 8, 24 | 5.0 |
|  |  | R | 54, 14, 26 | 4.8 |
| **Task – ITI** | | | | |
| Conjunction  (Sudoku/RDM) | inferior frontal junction (IFJ) | L | -46, 4, 30 | 4.2 |
|  |  | R | 52, 6, 28 | 4.4 |
| **Task – control during the redecision phase** | | | | |
| Conjunction  (Sudoku/RDM) | lateral frontopolar cortex (lFPC) | L | -30, 52, 6 | 4.7 |
|  |  | R | 34, 56, 2 | 3.0 |
|  | dorsolateral prefrontal cortex (DLPFC) | L | -46, 30, 22 | 3.8 |
|  |  | R | 46, 36, 22 | 4.8 |
|  | dorsal anterior cingulate cortex (dACC) | – | -2, 10, 44 | 5.8 |
|  | anterior insular cortex (AIC) | L | -30, 26, -8 | 6.1 |
|  |  | R | 32, 26, -10 | 6.5 |
|  | anterior inferior parietal lobule (aIPL) | L | -32, -56, 44 | 5.6 |
|  |  | R | 34, -54, 48 | 5.6 |
|  | inferior frontal junction (IFJ) | L | -48, 8, 24 | 5.4 |
|  |  | R | 52, 12, 28 | 5.2 |
| **Redecision – No-redecision** | | | | |
| Conjunction  (Sudoku/RDM) | lateral frontopolar cortex (lFPC) | L | -30, 50, 10 | 3.2 |
|  | dorsolateral prefrontal cortex (DLPFC) | L | -44, 28, 22 | 3.1 |
|  |  | R | 48, 38, 20 | 4.3 |
|  | dorsal anterior cingulate cortex (dACC) | – | -4, 24, 36 | 4.7 |
|  | anterior insular cortex (AIC) | L | -32, 24, -6 | 5.6 |
|  |  | R | 32, 22, -6 | 5.3 |
|  | anterior inferior parietal lobule (aIPL) | L | -34, -46, 44 | 4.0 |
|  |  | R | 38, -48, 42 | 4.0 |
|  | inferior frontal junction (IFJ) | L | -44, 4, 26 | 4.6 |
|  |  | R | 52, 14, 26 | 5.1 |
