# Supplementary material for: The neural system of metacognition accompanying decision-making in the prefrontal cortex
Source: PLoS Biol. 2018 Apr 23;16(4):e2004037. doi: 10.1371/journal.pbio.2004037 (PMC5933819; doi:10.1371/journal.pbio.2004037)
Supplement: S2 Table — (DOCX) [file pbio.2004037.s003.docx]

S2 Table. Activations correlated with the uncertainty level and the uncertainty reduction during the redecision phase.

| **Task** | **Anatomical Region** | **Hemispheres** | **Coordinate**  **(x, y, z)** | **Maximum** |
| --- | --- | --- | --- | --- |
| **Uncertainty (positive)** | | | | |
| Conjunction  (Sudoku/RDM) | lateral frontopolar cortex (lFPC) | L | -30, 56, 4 | 4.2 |
|  |  | R | 30, 52, 10 | 3.5 |
|  | dorsolateral prefrontal cortex (DLPFC) | L | -44, 28, 24 | 4.4 |
|  |  | R | 42, 30, 22 | 3.7 |
|  | dorsal anterior cingulate cortex (dACC) | – | -4, 14, 46 | 4.8 |
|  | anterior insular cortex (AIC) | L | -30, 26, -4 | 4.3 |
|  |  | R | 32, 24, -2 | 4.5 |
|  | anterior inferior parietal lobule (aIPL) | L | -48, 12, 26 | 4.0 |
|  |  | R | 48, 14, 24 | 3.7 |
|  | anterior inferior parietal lobule (aIPL) | L | -32, -56 40 | 3.9 |
|  |  | R | 44, -42, 54 | 3.8 |
| **Uncertainty (negative)** | | | | |
| Conjunction  (Sudoku/RDM) | ventromedial prefrontal cortex (VMFPC) | – | 0, 48, -14 | 3.8 |
|  | posterior cingulate cortex (PCC) | – | 0, -48, 22 | 4.0 |
| **Uncertainty reduction (positive)** | | | | |
| Sudoku | ventral striatum  (VS) | L | -10, 12, -8 | 4.2 |
|  |  | R | 10, 12, -4 | 4.5 |
|  | ventromedial prefrontal cortex (VMFPC) | – | -2, 56, -4 | 4.2 |
