# Supplementary material for: The neural system of metacognition accompanying decision-making in the prefrontal cortex
Source: PLoS Biol. 2018 Apr 23;16(4):e2004037. doi: 10.1371/journal.pbio.2004037 (PMC5933819; doi:10.1371/journal.pbio.2004037)
Supplement: S3 Table — (DOCX) [file pbio.2004037.s004.docx]

S3 Table. Activations positively correlated with the individual uncertainty sensitivity and the individual accuracy change.

| **Task** | **Anatomical Region** | **Hemispheres** | **Coordinate**  **(x, y, z)** | **Maximum** |
| --- | --- | --- | --- | --- |
| **Uncertainty sensitivity (*A*_roc_)** | | | | |
| Sudoku | dorsal anterior cingulate cortex (dACC) | R | 8, 16,38 | 3.5 |
|  | anterior insular cortex (AIC) | L | -38, 22, -4 | 3.8 |
|  |  | R | 42, 24, -12 | 4.1 |
| RDM | dorsal anterior cingulate cortex (dACC) | L | -4, 18, 48 | 3.4 |
| **RT-uncertainty correlation coefficient^1^** | | | | |
| Sudoku | dorsal anterior cingulate cortex (dACC) | R | 6, 18,42 | 3.1 |
| **Mean uncertainty** | | | | |
| Sudoku | dorsal anterior cingulate cortex (dACC) | L | -6, 16,44 | 3.3 |
| RDM | dorsal anterior cingulate cortex (dACC) | R | 4, 14, 46 | 3.6 |
|  | anterior insular cortex (AIC) | R | 40, 22, -14 | 3.5 |
| **Accuracy change** | | | | |
| Sudoku | lateral frontopolar cortex (lFPC) | L | -26, 54, 10 | 4.0 |
|  |  | R | 26, 48, 6 | 3.7 |

^1^after orthogonalization with the uncertainty sensitivity.
