# Supplementary figures and images for: The neural system of metacognition accompanying decision-making in the prefrontal cortex
Source: PLoS Biol. 2018 Apr 23;16(4):e2004037. doi: 10.1371/journal.pbio.2004037 (PMC5933819; doi:10.1371/journal.pbio.2004037)

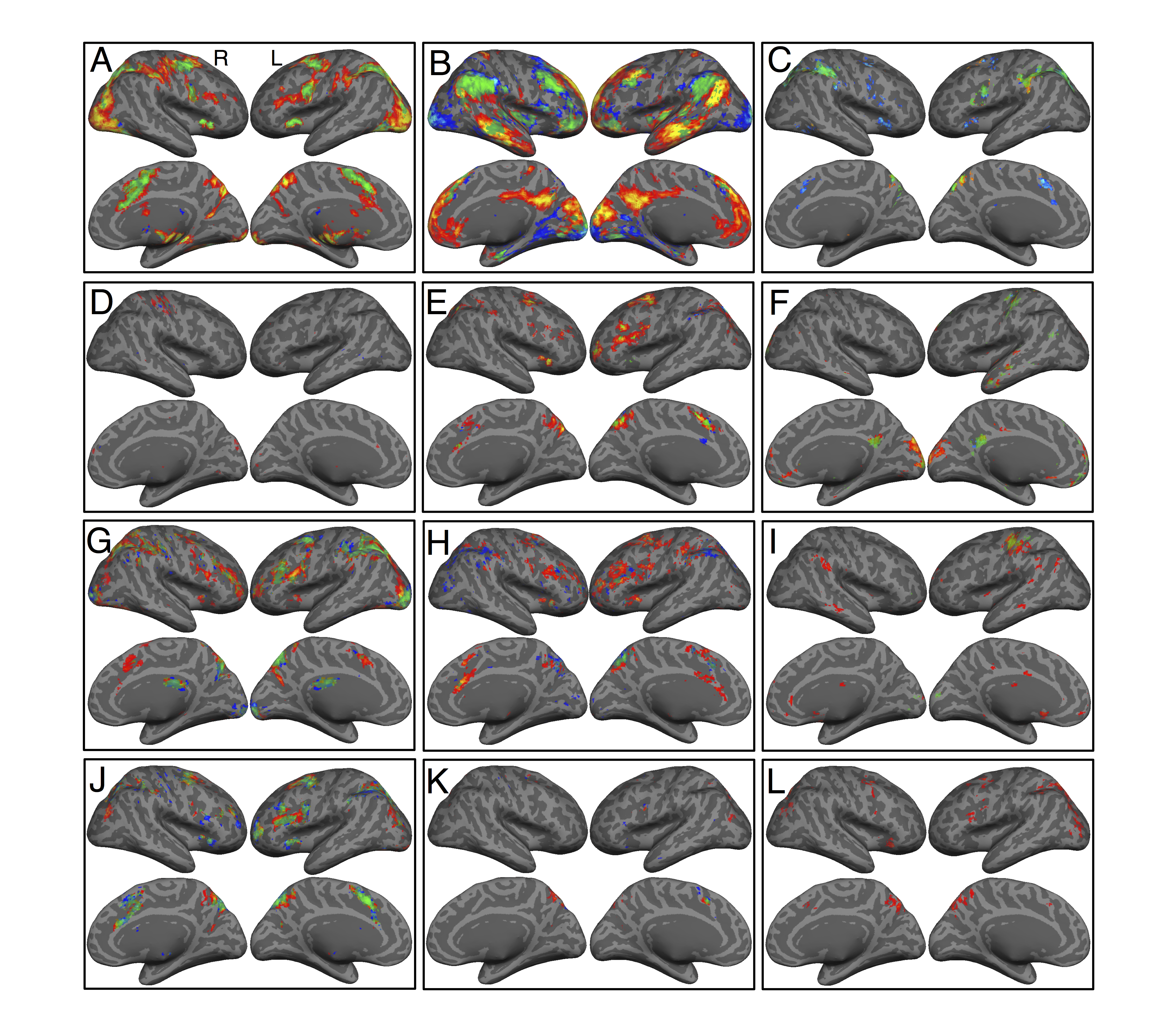

Supplement: S1 Fig — (A) Activations during the decision phase compared with those during the ITI period in fMRI1. (B) Activations during the redecision phase compared with those during the decision phase in fMRI1. (C) Activations of the initial decision during the redecision phase compared with those of the control condition during the same phase in fMRI2. (D) Positive correlation of activity during the decision phase with the decision uncertainty level in fMRI1 (there were also no negative correlations). (E) Positive correlation of activity during the redecision phase of the correct trials with the decision uncertainty level of the initial decision in fMRI1. (F) Negative correlation of activity during the redecision phase with the decision uncertainty level of the initial decision in fMRI1. (G) Activations during the redecision phase without requirement to decide the previous situation again (‘non-redecision’ condition) compared with those of the control trials during the same phase in fMRI3. (H) Positive correlation of activity during the redecision phase with the level of decision uncertainty reduction in fMRI1. (I) Positive correlation of activity during the redecision phase with the level of decision uncertainty reduction after orthogonalization with the decision uncertainty level in fMRI1. (J) Positive correlation of activity during the redecision phase with the decision uncertainty level after orthogonalization with the level of decision uncertainty reduction in fMRI1. (K) Negative correlation of activity during the redecision phase with the level of decision uncertainty reduction after orthogonalization with the decision uncertainty level in fMRI1. (L) Positive correlation of activity during the redecision phase with the interaction between the decision uncertainty level and the level of decision uncertainty reduction in fMRI1. The conventions are the same as in Fig 3. fMRI, functional magnetic resonance imaging; ITI, intertrial interval. (TIF) [file pbio.2004037.s005.tif]

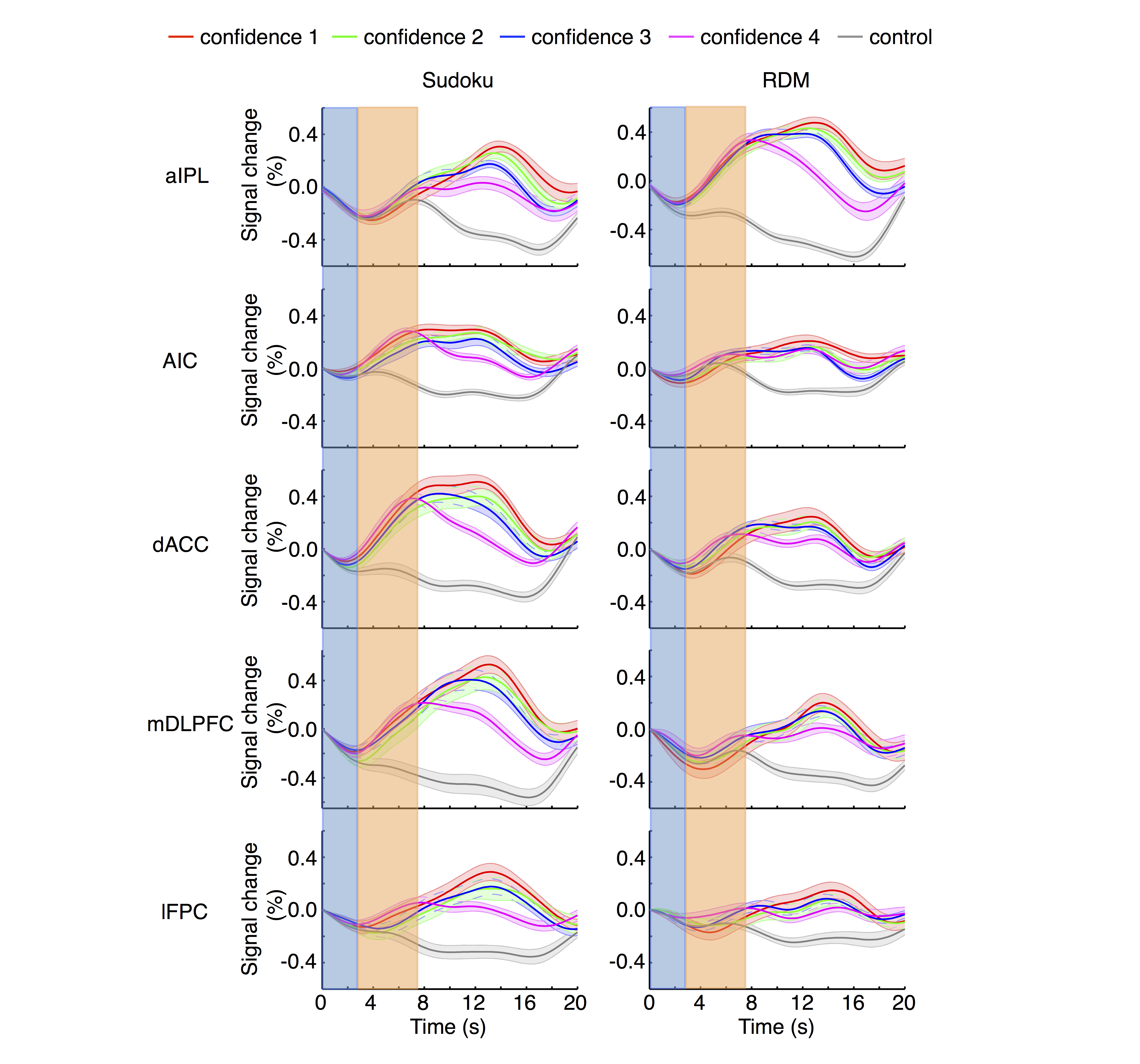

Supplement: S2 Fig — The time zero was the onset of the initial decision. The blue shadow represents the initial decision-making period, and the yellow shadow represents the redecision period. The data can be found in S1 Data. AIC, anterior insular cortex; aIPL, anterior inferior parietal lobule; dACC, dorsal anterior cingulate cortex; fMRI, functional magnetic resonance imaging; lFPC, lateral frontopolar cortex; mDLPFC, middle dorsolateral prefrontal cortex. (TIF) [file pbio.2004037.s006.tif]

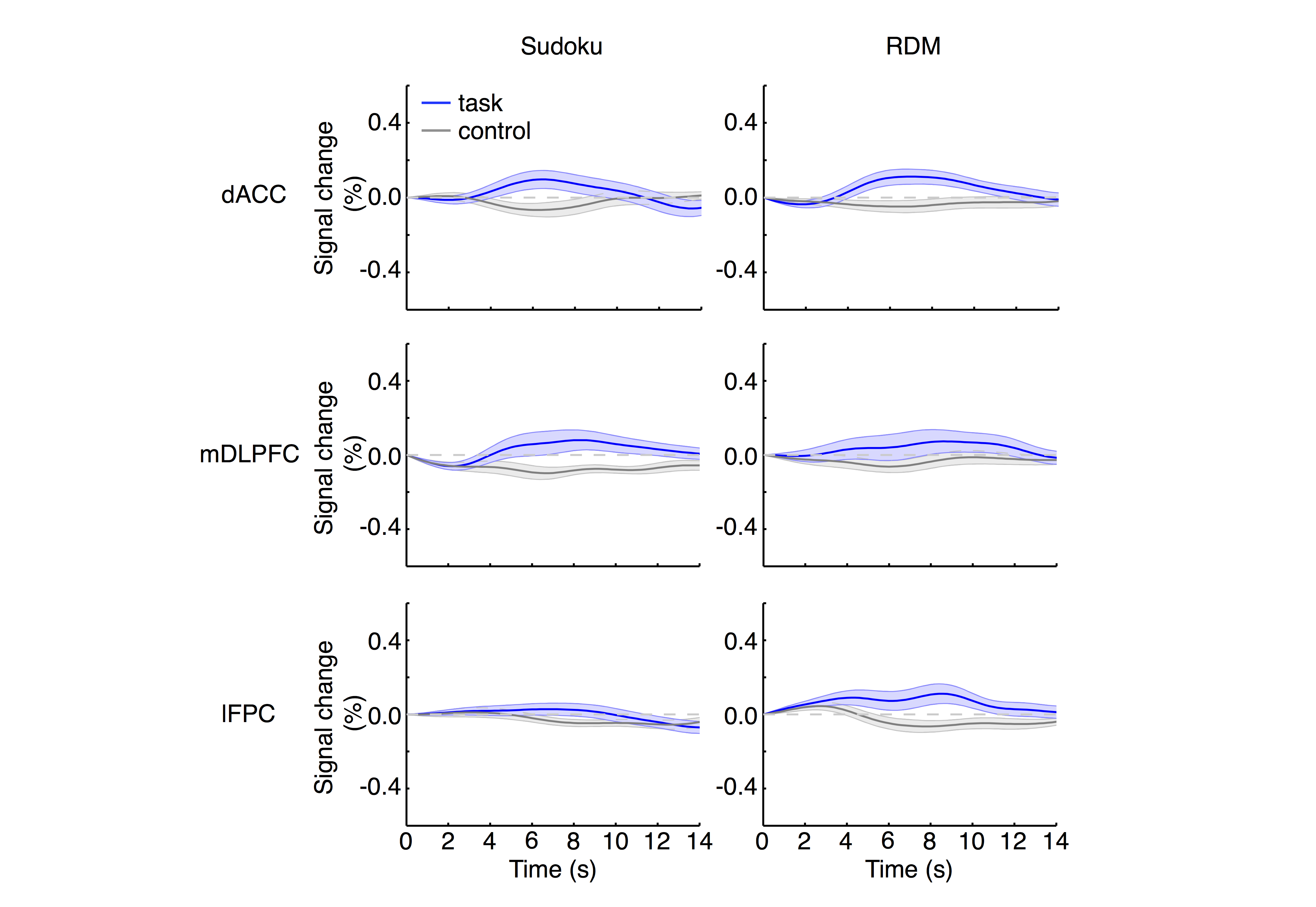

Supplement: S3 Fig — The time zero was the onset of the stimulus presentation in the second phase. The participant made the initial decision in the second phase, and the decision duration lasted for 4 s, longer than the initial decision period (2 s) in fMRI1. It should be noted that there was no significant activity in the mDLPFC and lFPC in the task trials, whereas the weak dACC activity in the task trials was delayed for over 3 s from the onset of the stimulus presentation. The data can be found in S1 Data. dACC, dorsal anterior cingulate cortex; fMRI, functional magnetic resonance imaging; lFPC, lateral frontopolar cortex; mDLPFC, middle dorsolateral PFC. (TIF) [file pbio.2004037.s007.tif]

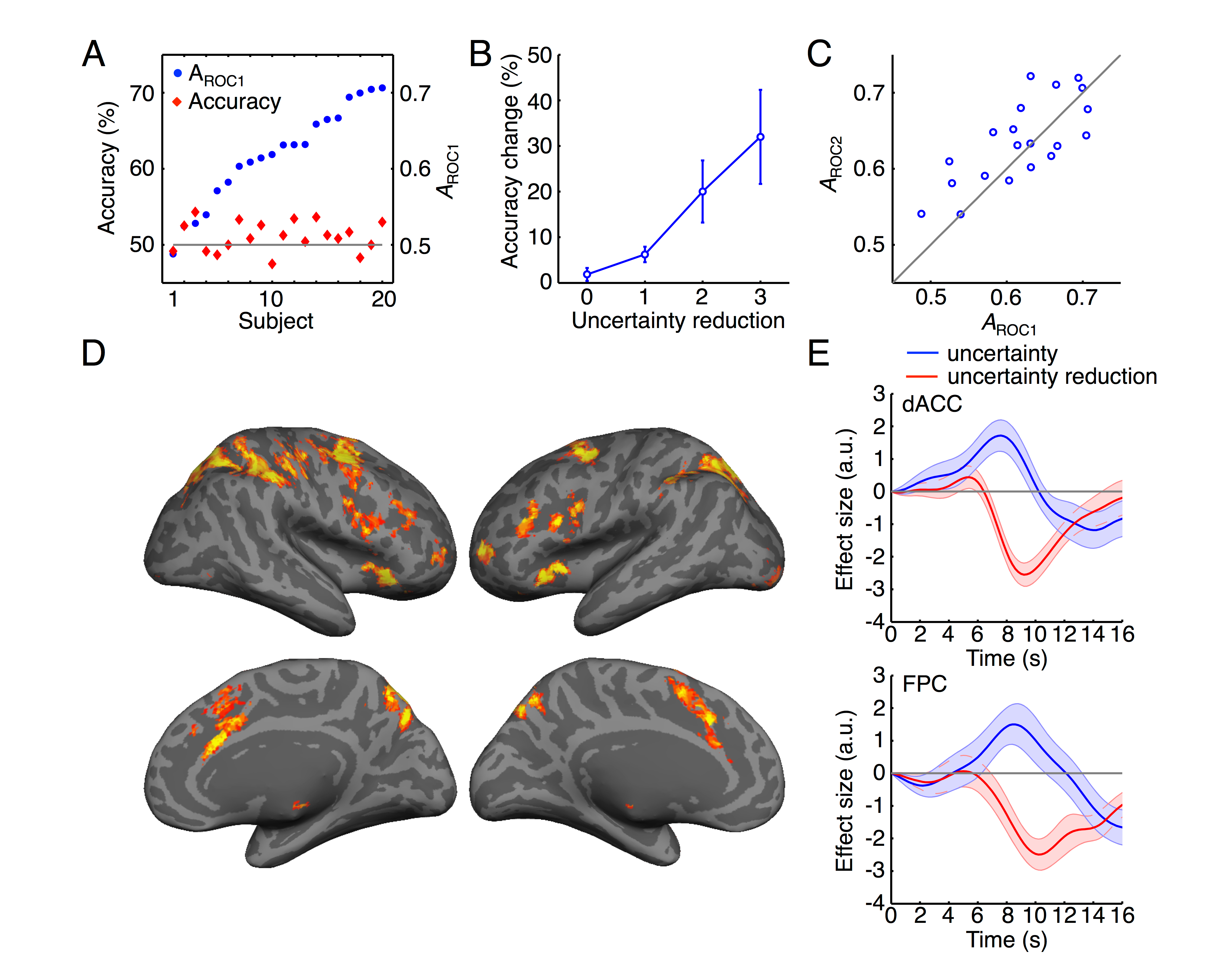

Supplement: S4 Fig — (A) The individual uncertainty sensitivity (AROC, blue circles) and decision accuracy (red diamonds) in the initial decision. (B) The relationship between the extent of decision uncertainty reduction and the accuracy change by redecision. (C) The individual uncertainty sensitivity (AROC) in the initial and final decisions. There was no difference between the two uncertainty sensitivities (t19 = 1.3, P = 0.10). (D) The z-statistic activation map of the task trials in comparison with those of the control trials during the redecision phase. z = 3.1, P < 0.05, FDR correction. (E) The dACC and FPC activity was positively correlated with the decision uncertainty level and negatively correlated with the extent of decision uncertainty reduction. The data can be found in S1 Data. dACC, dorsal anterior cingulate cortex; FDR, false discovery rate; fMRI, functional magnetic resonance imaging; FPC, frontopolar cortex. (TIF) [file pbio.2004037.s008.tif]
